# A New Basal Caniform (Mammalia: Carnivora) from the Middle Eocene of North America and Remarks on the Phylogeny of Early Carnivorans

**DOI:** 10.1371/journal.pone.0024146

**Published:** 2011-09-14

**Authors:** Susumu Tomiya

**Affiliations:** Museum of Paleontology, Museum of Vertebrate Zoology, and Department of Integrative Biology, University of California, Berkeley, California, United States of America; University College London, United Kingdom

## Abstract

**Background:**

Despite a long history of research, the phylogenetic origin and initial diversification of the mammalian crown-group Carnivora remain elusive. Well-preserved fossil materials of basal carnivorans are essential for resolving these issues, and for constraining the timing of the carnivoran origin, which constitutes an important time-calibration point in mammalian phylogenetics.

**Methodology/Principal Findings:**

A new carnivoramorphan from the middle Eocene of southern California, *Lycophocyon hutchisoni*, is described. The new taxon exhibits stages of dental and basicranial evolution that are intermediate between earlier carnivoramorphans and the earliest representatives of canoid carnivorans. The evolutionary affinity of the new taxon was determined by a cladistic analysis of previously-published and newly-acquired morphological data for 30 Paleogene carnivoramorphans. The most-parsimonious trees identified *L. hutchisoni* as a basal caniform carnivoran, and placed (1) *Tapocyon robustus*, *Quercygale angustidens*, “*Miacis*” *sylvestris*, “*M.*” *uintensis*, and “*M.*” *gracilis* inside or outside the Carnivora, (2) nimravids within the Feliformia, and (3) the amphicyonid *Daphoenus* outside the crown-group Canoidea. Parsimony reconstructions of ancestral character states suggest that loss of the upper third molars and development of well-ossified entotympanics that are firmly fused to the basicranium (neither condition is observed in *L. hutchisoni*) are not associated with the origin of the Carnivora as traditionally thought, but instead occurred independently in the Caniformia and the Feliformia. A discriminant analysis of the estimated body weight and dental ecomorphology predicted a mesocarnivorous diet for *L. hutchisoni*, and the postcranial morphology suggests a scansorial habit.

**Conclusions/Significance:**

*Lycophocyon hutchisoni* illuminates the morphological evolution of early caniforms leading to the origin of crown-group canoids. Considerable uncertainty remains with respect to the phylogenetic origin of the Carnivora. The minimum date of caniform-feliform divergence is provisionally suggested to be either 47 million years ago or 38 million years ago, depending on the position of “*Miacis*” *sylvestris* within or outside the Carnivora, respectively.

## Introduction

The first major effort to reconstruct the ancestry of the mammalian order Carnivora goes back over a century. As early as 1898, Scott [1] took particular note of numerous skeletal similarities among carnivorans from the late Eocene to early Oligocene of western North America, such as the nimravid *Dinictis*, the amphicyonid *Daphoenus* (then regarded as a canid), and the canid *Hesperocyon*. He interpreted these similarities as an indication for basal divergences of carnivoran lineages not long before the Oligocene. Around the same time, Wortman and Matthew [2], working on the systematics of carnivoramorphans (carnivorans and their close relatives) from the middle-Eocene Bridger and Uinta Formations of Wyoming and Utah, inferred largely linear series of descent from such fossil taxa as *Uintacyon* and *Procynodictis* to some of the extant canids based on what they recognized as progressive stages of skeletal evolution. Matthew [3] later expanded upon this study and presented a more complex phylogeny, portraying the early radiation of carnivoramorphans as divergent adaptations to various habitats and diets. His monumental work was soon followed by that of Teilhard de Chardin [4] on early carnivorans from the Eocene-Oligocene fissure-fill deposits of Quercy, France. Through a detailed study of dental morphology, Teilhard proposed that many of the lineages leading to extant families had already separated by the Miocene. These early workers were keenly aware of the difficulty of distinguishing phylogenetically-informative traits from parallel or convergent similarities, but lacked an analytical framework to deal with this problem.

The introduction of cladistics in paleontology thus provided an impetus for renewed investigations of the carnivoran origin, and precipitated in the last 30 years the seminal works of Flynn and Galiano [5], Wang and Tedford [6], and most recently, Wesley-Hunt and Flynn [7]. Respectively, these studies advanced foundational hypotheses on carnivoramorphan clades [5], unraveled the intricacies of basicranial evolution from early carnivoramorphans to early canids [6], and clarified the relationships of some of the basal carnivoramorphan groups to carnivorans [7]. Still, a holistic understanding of the phylogenetic, biogeographic, and ecological context of the carnivoran origin has yet to emerge, owing to the paucity of well-preserved basicranial and postcranial remains for many of the Paleogene taxa, as well as the limited spatial sampling of fossils both at the continental and global scales [8], [9].

This paper presents a taxonomic description of a new genus of carnivoramorphan from the Eocene Epoch, which constitutes a critical period of major cladogenetic events within the Carnivora [10]–[12]. Cladistic analyses were conducted to assess the phylogenetic affinity of the new taxon and to further elucidate the evolutionary relationships among early carnivorans and their close carnivoramorphan relatives. In addition, the diet and locomotor habit of the new taxon are discussed to facilitate future studies of carnivoramorphan evolution from the ecological perspective.

In this paper, I follow Bryant's ([13]:p. 184) phylogenetic definitions of higher taxa emended from Wyss and Flynn [14]: the crown-group Carnivora is defined as the “most recent common ancestor of Feloidea, all species referred to Canidae by Wilson and Reeder [15], and Arctoidea and all of its descendants”; the name Carnivoramorpha is applied to the more inclusive, stem-based group consisting of the “Carnivora and all members of Mammalia [16] that are more closely related to Carnivora than to taxa referred to Creodonta by Carroll [17].” It should be noted, however, that the sister-group relationship of the Carnivora and Creodonta is yet to be demonstrated in a comprehensive cladistic study of eutherian mammals [18]. Phylogenetically, the origin of Carnivora is the point of divergence of its two major lineages, the Caniformia and the Feliformia. Within the stem-group Caniformia, the crown group Canoidea encompasses the “most recent common ancestor of Arctoidea and the species referred to Canidae by Wilson and Reeder [15] and all of its descendants” ([13]:p. 184).

Accurate estimates of lineage divergence dates are essential for studies of trait evolution [19], diversity dynamics and biogeographic histories of major groups [12], [20]–[23], and ecological community assembly [24], as well as for the evaluation of biological conservation priorities [25], [26]. The node that marks the caniform-feliform divergence is important in mammalian phylogenetics because it is frequently selected as one of multiple fossil calibration points used in deriving the time scale for a molecular tree [20], [27]–[39]. Judicious selection of a fossil constraint in this context requires the knowledge of cladistic relationships of relevant fossil taxa, and must be updated according to the advancement of phylogenetic hypotheses in paleontology.

### Geographical and Geological Context

**Figure 1:**
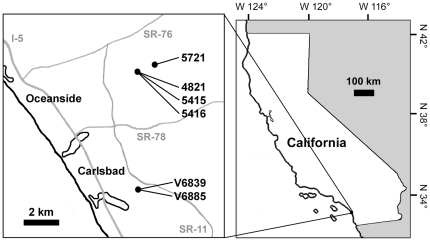
Map of localities that have yielded specimens of *Lycophocyon hutchisoni*. Localities with the prefix “V” are UCMP localities, and the rest are SDSNH localities.

All currently-known specimens of the new carnivoramorphan come from the middle-Eocene non-marine sediments of “member C” (an informally-designated unit) [40] of the Santiago Formation in San Diego County, California (Fig. 1). The holotype and a paratype (UCMP 170713) were collected in 1968 by personnel of the University of California Museum of Paleontology (UCMP; Berkeley, California, U.S.A.) at the Laguna Riviera housing subdivision in Carlsbad, California. Golz [41] reported the lithology of the holotype locality, V6839, as successive layers of sand and mudstone, in which most of the vertebrate fossils were concentrated in the sand-mud transitional zone. Based on this and the occurrence of reed impressions and brackish to freshwater invertebrates in the mudstone, he interpreted the depositional environment for the vertebrate remains to have been transitional between fluvial and lagoonal. The locality V6885, which yielded UCMP 170713, is a small sedimentary pocket of sandstone with a high concentration of vertically-oriented skeletal elements, and is located roughly 2 meters below the level of V6839 (D.P. Whistler, field notes for August 8, 1968, on file at the UCMP).

Golz [41] described the mammalian assemblages from V6839 and other localities in its vicinity as the Laguna Riviera Local Fauna, and considered them to be of the late Uintan North American Land Mammal Age (NALMA) based on the occurrence of the leporid *Mytonolagus* and the composition of artiodactyls similar to that in the Myton Member of the Uinta Formation, Utah. However, in the most-recent summary of middle-Eocene mammalian assemblages from San Diego County, Walsh [42] suggested the possibility of an early Duchesnean NALMA for the Laguna Riviera Local Fauna based partly on the occurrence of the rhinocerotoid *Amynodontopsis bodei* and the pantolestan *Simidectes merriami*.

The remaining specimens are from the San Diego Natural History Museum (SDSNH; San Diego, California, U.S.A.) localities at the Ocean Ranch Corporate Centre, Oceanside, California. Most of the localities are associated with sandy channel-deposits, and all are assigned to the Duchesnean NALMA based on the taxonomic composition of mammals [43].

A diverse array of vertebrate taxa are known from the holotype locality V6839, including fish, turtles, snakes, crocodiles, and birds. The mammalian component of the assemblage is numerically dominated by small to medium-sized selenodont artiodactyls such as *Leptoreodon* and *Protoreodon*, but also includes: erinaceomorph lipotyphlans; ischyromyid, cylindrodontid, and dipodid rodents; omomyid primates; and members of the enigmatic groups Apatotheria (*Apatemys* sp.) and Pantolesta (*Simidectes merriami*).

## Results

### Systematic Paleontology

Mammalia sensu Rowe, 1988 [16]

Carnivoramorpha sensu Bryant, 1996 [13]

Carnivora sensu Bryant, 1996 [13]

Caniformia sensu Bryant, 1996 [13]

Family-group indet.

***Lycophocyon*** **, gen. nov.**

urn:lsid:zoobank.org:act:CCD7EEE0-1EE9-4C73-A207-3F2CB9C49F42

#### Type species

*Lycophocyon hutchisoni*, gen. et sp. nov.

#### Diagnosis

As for type species.

#### Etymology

From the Greek λυκόφως, twilight, and κυόν, dog; in references to its occurrence on the west coast of North America, and its probable affiliation with caniform carnivorans.

#### Distribution

As for type species.

***Lycophocyon hutchisoni*** **, gen. et sp. nov.**

urn:lsid:zoobank.org:act:7186E061-58AC-49D6-8499-CDF41ECF21FC

#### Diagnosis

Differs from canoid carnivorans in absence of well-ossified entotympanics that are firmly attached to basicranium. Differs from other non-canoid carnivoramorphans in broad and flat anterior extension of petrosal promontorium. Further differs from: (1) both amphicyonids and canids in greater anterolabial extension of M1 parastylar region such that distance between paracone and anterolabial tooth margin roughly equals distance between paracone and protocone, and M1 posterior lingual cingulum that is not as raised as protocone; (2) arctoids in swelling of M1 posterior lingual cingulum (though not as raised as in amphicyonids and canids), and presence of M3; (3) feliforms in presence of unreduced postglenoid foramen, presence of unreduced M1, presence of M3, and presence of moderately-developed m1 talonid; (4) “*Miacis*” *cognitus* in P3 with well-defined posterior accessory cusp, greater anterolabial extension of M1 parastylar region, and more reduced M2; (5) both *Procynodictis* and “*Miacis*” *gracilis* in having proportionately longer M1 (M1L/M1W>0.60), less-developed cuspulids on anterior and posterior cingulids of p3 and p4, and more lingually-directed m1 paraconid (giving trigonid more closed appearance); (6) *Procynodictis* in more rounded anterolabial corner of P4, and more posterior placement of M1 protocone; (7) “*M.*” *gracilis* in anterior tilt of M1 parastylar region, more reduced M2 protocone, and less-pronounced lingual protrusion of m1 metaconid; (8) “*Miacis*” *uintensis* in having p4 that is shorter than m1 (p4L≥m1L in “*M.*” *uintensis*) and more straight posterior slope of p4 owing to less-developed cuspulid on posterior cingulid; (9) “*Miacis*” *sylvestris* in larger size (m1>20% longer), presence of posterior accessory cusp/cuspid on P3 and p4, better-developed posterior lingual cingulum of M1, more reduced m2 trigonid cuspids, more reduced and simplified m3, and absence of sulci on petrosal promontorium for promontory and stapedial branches of internal carotid artery; (10) *Miacis parvivorus* in larger size (m2>25% longer), greater anterolabial extension of M1 parastylar region, more triangular outline of M1 in occlusal view, and m1 and m2 with more open trigonid; (11) *Quercygale* in wide shelf between mastoid process and paroccipital process that does not form a trough, better-developed M1 posterior lingual cingulum, and presence of M3; (12) *Tapocyon* in less-pronounced labial extension of M1 parastylar region, larger M2 relative to M1 (M2W/M1W>0.60), presence of M3, larger m1 talonid relative to trigonid, less-developed cuspulid on posterior cingulid of p4, larger m2 relative to m1 (m2L/m1L>0.55), and more gradual tapering of dentary toward its anterior end; (13) *Dawsonicyon* in larger size (m1>40% longer) and p4 with more dorsally-positioned posterior accessory cuspid; (14) viverravids in having M1 with protocone that is shorter than paracone, presence of M3 and m3, and low trigonid and short talonid of m2; (15) all other known carnivoramorphans in the combination of: well-ossified tegmen tympani; petrosal promontorium in medial contact with basioccipital; slight ventral deflection of ventral floor of basioccipital along middle ear chamber; absence of sulci on petrosal promontorium for promontory and stapedial branches of internal carotid artery; P3, p3, and p4 with well-defined posterior accessory cusp/cuspid located between main cusp and posterior cingulum/cingulid; M1 and M2 with pronounced anterolabial extension of parastylar region; M1 protocone located near anterolingual border of tooth; M1 anterior lingual cingulum forming very thin band rather than shelf; crescentic M1 posterior lingual cingulum that is at least twice as wide in occlusal view as anterior lingual cingulum; M2 approximately one-third to one-half the size of M1 (when measured as the product of length and width in occlusal view); M2 and M3 with increasingly-reduced occlusal surficial relief; presence of diminutive M3; cuspulid on anterior cingulid of p2-p4 small or absent; p4 shorter than m1; and gradual tapering of dentary toward its anterior end.

#### Etymology

Specific name after J. Howard Hutchison, who led a UCMP team in a 1968 excavation that yielded the holotype and a paratype (UCMP 170713), and in honor of his contribution to the study of fossil vertebrates of California.

#### Distribution

Upper portions of “member C” [40] of the Santiago Formation, San Diego County, California, corresponding to the early Duchesnean and possibly also to the late Uintan NALMAs [41]–[43].

#### Holotype

UCMP 85202, right dentary fragment with p2-m1, left dentary with p2-m2, and cranial fragments with right P4-M2 and left P3-M2.

#### Holotype locality

UCMP locality V6839, Laguna Riviera 1, Santiago Formation, member C, Carlsbad, San Diego County, California, U.S.A.

#### Paratypes

UCMP locality V6885, Half-day Pocket, Santiago Formation, member C, Carlsbad, San Diego County, California, U.S.A.: UCMP 170713, right dentary with c1, p2-m2, left dentary fragments with c1, p1, m1, and cranial fragment with right P2, P4-M3.

SDSNH locality 5416, Ocean Ranch Phase 2C Bone Sands, Santiago Formation, member C, Oceanside, San Diego County, California, U.S.A.: SDSNH 107658, right dentary with m1-m3; SDSNH 107659, cranium with right P2, P4-M2, and left P2-M2.

SDSNH locality 5721, Ocean Ranch Phase 1B, Santiago Formation, member C, Oceanside, San Diego County, California, U.S.A.: SDSNH 107442, articulated cranium and mandible; SDSNH 107443, cranium with right P2-M2 and left P3-M1; SDSNH 107444, cranium with left P4-M2, left dentary fragments with c1, p2-p3; SDSNH 107446, cranium, dentary, caudal vertebra, left ulna, left femur, right tibia, right astragalus, middle phalanx; SDSNH 107447, left dentary with p1-m1, left humerus.

#### Referred specimens

UCMP locality RV6830 (same quarry as UCMP locality V6839 [41]), Laguna Riviera Quarry, Santiago Formation, member C, Carlsbad, San Diego County, California, U.S.A.: UCMP 313994, left m1.

SDSNH locality 4821, Rancho Del Oro Road Extension, Santiago Formation, member C, Oceanside, San Diego County, California, U.S.A.: SDSNH 92094, right dentary with p2-m1, left dentary with c1-p4.

SDSNH locality 5415, Ocean Ranch Phase 2A Bone Sands, Santiago Formation, member C, Oceanside, San Diego County, California, U.S.A.: SDSNH 105783, right dentary with c1, p4-m2.

SDSNH locality 5721, Ocean Ranch Phase 1B, Santiago Formation, member C, Oceanside, San Diego County, California, U.S.A.: SDSNH 107448, left dentary with p2-m2; SDSNH 107449, right dentary with c1, p2-m2; SDSNH 107450, left dentary with c1, p2-m2; SDSNH 107452, left dentary with c1-m1; SDSNH 107453, left dentary with m2; SDSNH 107455, left dentary fragment with m1; SDSNH 107456, right dentary; SDSNH 107457, left dentary with m1 and m2; SDSNH 107458, right dentary with p2-m1; SDSNH 107460, left dentary with c1-p4; SDSNH 107461, left dentary with c1-m2; SDSNH 107462, right P4; SDSNH 107465, edentulous cranium, right dentary with m2; SDSNH 107467, edentulous left dentary; SDSNH 107468, edentulous left dentary; SDSNH 107538, partial cranium.

#### Remarks

*Lycophocyon hutchisoni* is here classified as a caniform carnivoran based on the result of a cladistic analysis in the present study, as discussed below. The familial affiliation of *L. hutchisoni* is indeterminate with the current knowledge of the species and basal carnivoran phylogeny.

### Description

Unless otherwise noted, the description of the cranium is based on the holotype UCMP 85202. The descriptions of caudal vertebra, ulna, femur, tibia, astragalus, and intermediate phalanx are based on the paratype SDSNH 107446 with an associated skull, and the description of humerus is based on the paratype SDSNH 107447 with an associated left dentary fragment with p1-m1 that can be confidently identified as belonging to *Lycophocyon hutchisoni*. Craniodental and postcranial measurements are reported in Table 1 and Table 2, respectively. A list of comparative specimens directly examined by the author is provided in Appendix S1. Comparisons with published accounts and figures of other taxa should be considered preliminary. References to character numbers pertain to the cladistic analysis discussed below; the characters, character states, and their numbering follow those of Wesley-Hunt and Flynn [7].

**Table 1:** Craniodental measurements (in mm) of *Lycophocyon hutchisoni*.

Measurement	UCMP 85202	UCMP 170713	SDSNH 107442	SDSNH 107443	SDSNH 107444	SDSNH 107447	SDSNH 107465	SDSNH 107658	SDSNH 107659
**Cranium**									
Length^1^			∼105				105.3		
W_int_									∼31
WC1									18.4
WM1									34.1
**Mandible**									
Length^2^	98.7		∼91						
Dm1	16.1					16.5		13.6	
**Dentition**									
I1W			∼1.3						
I2W			∼1.2						
I3L			∼3.3						
C1L			∼7.0						
P2L		5.5	6.4	6.0					5.6
P2W		2.5		2.6					2.4
P3L	6.7		6.7	7.6					6.4
P3W	4.3			4.3					3.5
P4L	10.9	10.3	10.6	10.5					9.4
P4W	7.6	7.4		7.6					6.4
M1L	8.1	6.8	∼8	7.4	6.8				6.5
M1W	11.9	10.9		11.4	11.0				9.9
M2L	4.9	3.8		4.3	4.6				4.5
M2W	8.0	6.6		7.7	7.6				7.2
M3L		1.8							
M3W		2.9							
c1L		5.5	∼5.8						
c1W		3.4	∼4.1						
p1L		3.2				2.4			
p1W		1.8				2.0			
p2L	5.8	5.0	5.5			5.5			
p2W	2.6	2.4				2.6			
p3L	7.0	6.5	7.6			7.2			
p3W	3.5	3.2				3.1			
p4L	8.3	8.1	8.7			8.9			
p4W	4.3	3.9				3.8			
m1L	10.7	9.6				9.5			
m1W	6.5	5.7				6.2			
m2L	6.8	5.4	∼7					6.3	
m2W	4.9	4.1						4.1	
m3L								3.2	
m3W								2.6	

1 Length from the anterior end of premaxilla to the posterior end of occipital condyle.

2 Length from the anterior end of c1 alveolus to the posterior end of mandibular condyle. Where applicable, dental measurements are the arithmetic means of the right and left teeth.

**Abbreviations:** **Dm1**, depth below m1; **L**, anteroposterior length; **W**, labiolingual width; **WC1**, rostral width between labial margins of right and left C1; **W_int_**, interorbital width; **WM1**, palatal width between labial margins of right and left M1.

**Table 2:** Postcranial measurements (in mm) of *Lycophocyon hutchisoni*.

Measurement	SDSNH 107446	SDSNH 107447
**Caudal vertebra**		
TL	35.2	
MW	8.0	
**Humerus** 1		
HD		∼9.2
HDAB		∼21.1
HDAP		∼11.5
HEB		28.9
HL		∼105.2
**Ulna** 1		
ULO	∼16.2	
UOD	∼10.0	
UPA	15.1	
**Femur**		
TL	134.4	
**Astragalus**		
PDL	20.4	
TW	13.0	
HW	10.4	
**Phalanx**		
TL	13.6	
MW	3.9	

1 Measurements and abbreviations follow [127].

**Abbreviations: HD**, minimum transverse diameter of diaphysis; **HDAB**, distal width of trochlea and capitulum combined; **HDAP**, anteroposterior depth of distal humerus; **HEB**, maximum mediolateral width of distal humerus; **HL**, maximum length; **HW**, mediolateral width of astragalar head; **MW**, transverse width at mid-length; **PDL**, proximodistal length of astragalus; **TL**, total length; **TW**, mediolateral width of astragalar trochlea; **ULO**, length of olecranon process; **UOD**, anteroposterior depth of olecranon process; **UPA**, anteroposterior depth measured from anconeal process and parallel to UOD.

#### Cranium

**Figure 2:**
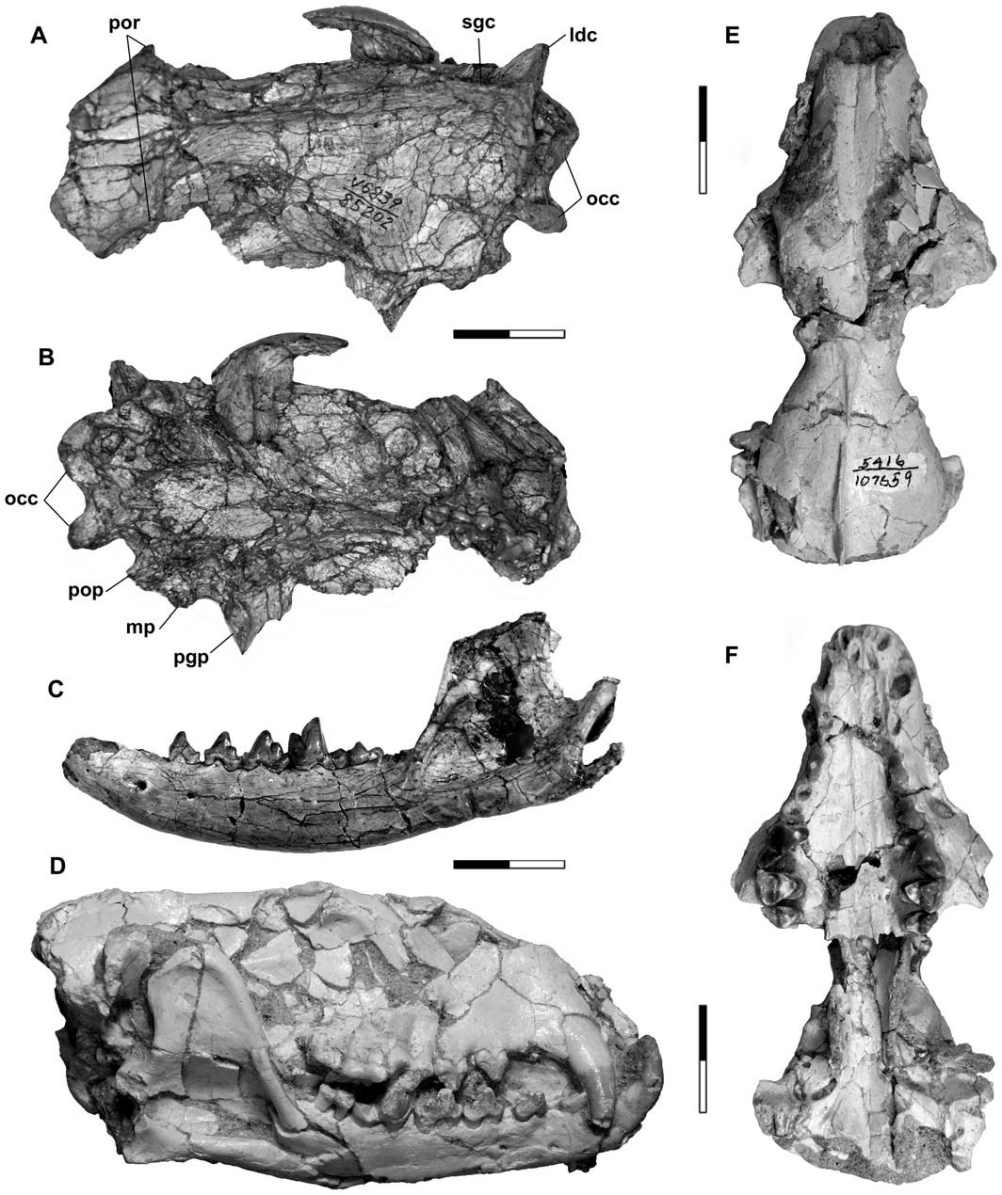
Crania and mandibles of *Lycophocyon hutchisoni*. Holotype UCMP 85202 (**A–C**), SDSNH 107442 (**D**), and SDSNH 107659 (**E, F**), showing cranium in dorsal (**A**) and ventral (**B**) views, left dentary (**C**), cranium articulated with mandible (**D**), and cranium in dorsal (**E**) and ventral (**F**) views. Scale bars equal 2 cm. **Abbreviations**: **ldc**, lambdoidal crest; **mp**, mastoid process; **occ**, occipital condyle; **pgp**, postglenoid process; **pop**, paroccipital process; **por**, postorbital process; **sgc**, sagittal crest.

**Figure 3:**
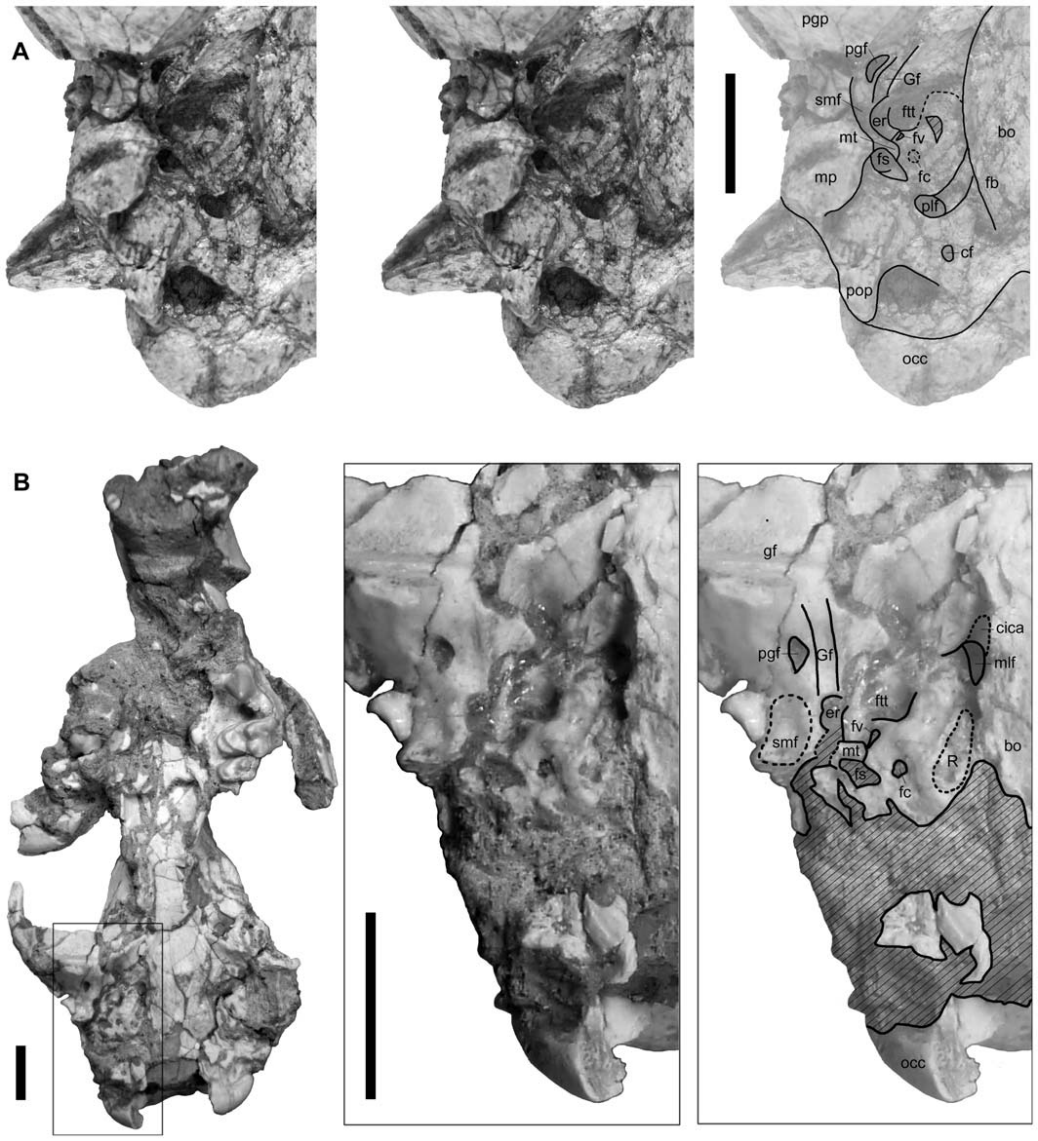
Right basicranial regions of *Lycophocyon hutchisoni*. Holotype UCMP 85202 (**A**, stereo pair) and SDSNH 107444 (**B**). **Abbreviations**: **bo**, basioccipital, **cf**, condyloid foramen; **cica**, canal for internal carotid artery; **er**, epitympanic recess; **fb**, lateral flange of basioccipital; **fc**, fenestra cochlea; **fs**, fossa for stapedius muscle; **ftt**, fossa for tensor tympani muscle; **fv**, fenestra vestibuli; **gf**, glenoid fossa; **Gf**, Glaserian fissure; **mlf**, middle lacerate foramen; **mp**, mastoid process; **mt**, mastoid tubercle; **occ**, occipital condyle; **pgf**, postglenoid foramen; **pgp**, postglenoid process; **plf**, posterior lacerate foramen; **pop**, paroccipital process; **R**, rugose area on petrosal promontorium; **smf**, suprameatal fossa. Scale bars equal 1 cm. Anterior to the top.

The crania of UCMP 85202 (Fig. 2A, B) and SDSNH 107659 (Fig. 2E, F) are missing the rostrum and much of the occipital region, respectively, and both are dorsoventrally crushed. The cranium of SDSNH 107442 (Fig. 2D) is nearly complete but crushed transversely. In all three specimens, frontals and parietals are fused. In SDSNH 107659, the sutures surrounding the pair of nasals are visible. The cranium of SDSNH 107444 (Fig. 3B) is missing much of the palate and the right maxilla, but preserves some details of the basicranium that are obscure in the holotype; the remaining bones are highly fragmented but largely held together by the sedimentary matrix.

While the type and referred specimens exhibit considerable craniodental size variation, the cranial length of *Lycophocyon hutchisoni* is comparable to those of such extant carnivorans as *Urocyon cinereoargenteus* (gray fox), *Martes pennanti* (fisher), and *Procyon lotor* (raccoon), and intermediate between those of the early canid *Hesperocyon gregarius* and the early amphicyonid *Daphoenus*. The rostrum (preorbital region) is wide and tall as in *M. pennanti* and *P. lotor* but proportionately longer (Fig. 2D–F). The braincase of *L. hutchisoni* is short (roughly 40% of the cranial length or smaller) and almost as narrow as the interorbital breadth measured between the anterior extremities of orbits (Fig. 2E). The dorsal border of braincase in profile is nearly horizontal in SDSNH 107442 (Fig. 2D). The cranial form in dorsal and ventral views closely resembles that of *Cynodictis lacustris* (cf. [4]:plate 2, figs. 1, 3), known from the late Eocene of Europe.

The premaxilla of SDSNH 107442 is short in dorsal aspect, and does not extend beyond the C1. The anterior end of the premaxilla bearing the alveoli for the upper incisors is mediolaterally highly compressed. Narrow incisive foramina are located immediately posterior to the I1 and I2, and extend slightly beyond the anterior margin of C1. In SDSNH 107443, the posterior border of premaxilla lateral to the incisive foramen is located next to the C1 (Character 2, state 0). The nasals maintain roughly the same width along most of their lengths, with tapered posterior extremities located above the anterior margins of the orbits (Character 63, state 0). Turbinal bones (Character 62) cannot be observed in any of the currently-known specimens. The maxilla is relatively long and bears a round (UCMP 85202) to dorsally-elongate (UCMP 170713) infraorbital foramen (Character 3; coded as state 0/elongate because UCMP 170713 appears to preserve the original shape more accurately) above the P3 (Character 4, state 0). The maxillary roof of the oral cavity is deeply excavated between the P4 and M1 to accommodate the relatively tall trigonid of M1 characteristic of early carnivoramorphans.

The pair of palatines forms a wedge-shaped anterior margin located as anteriorly as the protocone of P4. The midline-length of palatine is shorter than that of maxilla (19.6 mm and >29 mm, respectively, in SDSNH 107659; Character 60, state 0). The right and left tooth rows diverge gradually from their anterior ends to the posterior ends of P4s, such that the maximum palatal width is roughly 270% of the palatal width between the upper canines (Character 61, state 0). In SDSNH 107659, two openings of the palatine canal are discernible essentially along the left maxillopalatine suture (Character 6, state 1); the posterior end of palate on the median line is more or less aligned with the posterior end of the upper tooth row (Character 5, state 1). The extent of palatines on the lateral faces of the cranium (Character 65) is unclear.

The lacrimal is mostly broken and missing in the holotype, but is preserved intact in SDSNH 107659, showing a small exposure on the rostrum (lacrimal facial process; Character 1, state 1). The lacrimal foramen in UCMP 85202 is nearly circular and approximately 2 mm in diameter. In the holotype, the anterodorsal end of the jugal bears a probable contact surface with the lacrimal (Character 64, state 0). The large orbit bears a short, pointed postorbital process (Character 8, state 1), which gives rise to a ridge that connects to a well-delineated sagittal crest formed by the frontals and the parietals. The relative lengths of the frontal and the parietal are unclear because the fronto-parietal suture is apparently fused in all available crania (cf. Characters 7 and 66). In UCMP 85202 and SDSNH 107538, expansive lambdoidal crests are present. The zygomatic arch is particularly deep in UCMP 85202, suggesting the presence of a powerful masseter muscle. The large glenoid fossa is associated with a well-developed postglenoid process, but is laterally more open than in extant mustelids.

Morphological details of the basicranium (Fig. 3) are difficult to discern in the holotype because of poor preservation. The basisphenoid region is rather narrow, reflecting the constriction of the braincase. The fused basioccipital and basisphenoid form a somewhat fusiform floor. A pair of muscular tubercles presumably for the insertion of the longus capitis muscles is located at the posterolateral ends of this fusiform floor medial to the posterior lacerate foramina. In the early amphicyonids *Cynodictis* (cf. [44]:fig. 2, [45]:fig. 8) and *Daphoenus*, the fusiform floor terminates somewhat more anteriorly, and the muscular tubercles are correspondingly positioned medial to the petrosal promontoria. Although less pronounced than in *Hesperocyon gregarius*, the lateral edge of the ventral surface of the basioccipital shows slight ventral deflection (Character 34, state 1), and so it was likely in contact with the presumably unossified auditory bulla (see below). There is, however, no indication on the basioccipital and basisphenoid of pronounced medial inflation of the entotympanic as has been noted for some early and extant feliforms [7] (Character 35, state 0). Notably, the basioccipital bears a laterally-extended flange dorsal to the ventral floor. This flange is in contact with the medial face of promontorium, and forms a broad trough anterior to the posterior lacerate foramen (“foramen lacerum posterius primitivum” [44]). Similar basioccipital morphology has been reported for “*Miacis*” *sylvestris* (cf. [6]:fig. 3) and *Cynodictis* [44] (see also [45]:fig. 8), in which the flange presumably formed the roof of inferior petrosal sinus. The broad trough of *L. hutchisoni* (broader than that of “*M.*” *sylvestris*) may reflect an inferior petrosal sinus with a relatively large diameter. However, a very deep excavation of the basioccipital as in amphicyonids and ursids (known to accommodate a double-looped internal carotid artery in ursids [46]) seems unlikely because there is little vertical space, if any, between the promontorium and the underlying ventral floor of basioccipital in both the holotype and SDSNH 107659 (Character 31, state 0); in *Daphoenus* and *Ursus*, the promontoria are deeply (i.e. in the dorsal direction) embedded in the middle ear chambers relative to the level of the ventral floor of basioccipital.

Of the 4 known crania of *Lycophocyon hutchisoni* in which at least part of the middle-ear region can be observed, none preserves the auditory bulla, malleus, incus, or stapes. The bulla is therefore tentatively assumed to have been either made of a soft tissue or ossified but not as firmly attached to the basicranium as in more derived carnivorans (Character 68, state 0). Because no bulla is preserved, presence of an ectotympanic or entotympanic septum in the bulla cannot be determined (Characters 70 and 71). The petrosal promontorium (Fig. 3) is posterolaterally somewhat globular, and appears to have been medially in contact with the lateral edge of the ventral surface of basioccipital (Character 21, state 1). In SDSNH 107444, the promontorium is anteromedially elongate and flat (Fig. 3B; Character 28, state 3), resembling those of early canids and arctoids but differing from those of early amphicyonids with distinct, round anterior margins (cf. [45]:figs. 3, 9). The ventral surface of the promontorium in SDSNH 107444 is smooth except for a slightly rugose medial portion (“R” in Fig. 3B; Character 30, state 1). Rugose areas of similar extent in “*Miacis*” *sylvestris* and *Amphicticeps shackelfordi* have been interpreted as attachment areas for entotympanics [6], [47]. Unlike in earlier carnivoramorphans such as *Vulpavus profectus*, *Miacis parvivorus*, and “*M.*” *sylvestris* [6], the promontorium does not bear any arterial sulcus, suggesting an extrabullar passage of the internal carotid artery (Character 25, state 2), which is otherwise first known in *Hesperocyon gregarius* among caniform carnivorans [6]. The promontorium of *L. hutchisoni* resembles those of early arctoids such as *A. shackelfordi*, *Plesictis genettoides*, and *Broiliana nobilis* in having a moderately-expanded shelf posterior to the fenestra cochlea (Character 26, state 1); in contrast, the extent of this shelf is very limited in early amphicyonids such as *Daphoenus* and *Paradaphoenus*, presumably inheriting the primitive condition in carnivoramorphans [7] (see also [6]). Unlike in early feliforms such as *Palaeoprionodon lamandini*, *Stenogale julieni*, and *Proailurus lemanensis* [7], the promontorium of *L. hutchisoni* does not have a ventral process (Character 27, state 0) or a facet for the attachment of ectotympanic (Character 29, state 0). The fenestra vestibuli is elliptical, with the long axis pointing anteromedially. The similarly-sized fenestra cochlea (Character 72, state 0; clearly seen only in SDSNH 107444) is somewhat more circular in shape.

The deep fossa for stapedius muscle is approximately 2 mm in diameter, and is anteriorly bounded by the mastoid tubercle (Character 37, state 0). Similar size and depth characterize the clearly-demarcated posterior lacerate foramen (Character 17, state 1). The small, elongate condyloid foramen is located anterior to the groove between the occipital condyle and the paroccipital process (Character 16, state 1) and behind the posterior lacerate foramen such that their medial margins are more or less aligned; the latter two foramina are separated by a distance of more than the diameter of the condyloid foramen (Character 15, state 0). The mastoid tubercle is composed of the petrosal (Character 22, state 0), and the mastoid process is similar in size to the paroccipital process (Character 13, state 0). The precise orientation of the mastoid process (Character 14) is unclear because the extremity is missing on the left process, and the right process appears to have been ventrally reoriented distal to a breakage at its base. The mastoid tubercle in SDSNH 107444 is tightly appressed to the promontorium slightly anterior to the fenestra cochlea (Character 18, state 0). In comparison, the mastoid tubercles in *Daphoenus* are mediolaterally shorter and do not contact the promontoria, whereas those in *Cynodictis* are long and apparently lie ventral to the fenestra cochlea (cf. [44]:fig. 2, [45]:fig. 8). Because of poor preservation, it is unclear whether the mastoid tubercle of *Lycophocyon hutchisoni* bears an articular facet for the posterior limb of ectotympanic as in “*Miacis*” *cognitus*, *Miacis parvivorus*, and *Tapocyon robustus* [48], [49]. In the holotype and SDSNH 107444, a very shallow depression on the dorsal wall of the external auditory meatus appears to represent an incipient suprameatal fossa (Character 24, state 1) as in *Hesperocyon gregarius* [6], and is in contrast to the deep fossae in some of the early mustelidans such as *Plesictis genettoides* and *Broiliana nobilis*. No bony tube is preserved in association with the external auditory meatus, but the possibility of a tube formed by a cartilaginous bullar element cannot be discounted (Character 69).

The oblong postglenoid foramen is located lateral to the trough-like Glaserian fissure (but not near the lateral edge of skull; Characters 11 and 12, state 0), which, in turn, ascends steeply into the deeply-excavated epitympanic recess. There does not appear to be a deep, clearly-defined fossa on the squamosal for the contact with the anterior crus of ectotympanic (Character 32, state 0). The fossa for tensor tympani muscle is deep (Character 39, state 1). While the details are difficult to discern, there is no sign of an exposed canal for the facial nerve anterior to the promontorium, and it seems likely that the facial nerve was floored by the well-ossified tegmen tympani (Character 20, state 2). The promontory foramen cannot be identified in the available specimens. In SDSNH 107444, the middle lacerate foramen is anteriorly bounded by the tympanic wing of basisphenoid and posteriorly by the petrosal (Character 40, state 1); the tympanic wing of basisphenoid bears a depression with a well-delineated round anterior margin, suggesting the presence of an anterior loop of the internal carotid artery (Character 23, state 1). Presence of an epitympanic wing of the petrosal near the anteromedial corner of the fossa for tensor tympani muscle (Character 38) cannot be determined. In SDSNH 107659, the posterior opening of alisphenoid canal and the foramen ovale are respectively located at the anterior and the posterior ends of a groove (approximately 5 mm in length, 2 mm in width) behind the pterygoid, and are separated by a distance that is greater than the diameter of the alisphenoid opening (Character 19, state 0).

The long (∼9 mm in SDSNH 107465), pointed paroccipital process (Characters 9 and 10, state 0) is posteriorly-directed, and its ventral surface appears flat, resembling that of the early arctoid *Amphicticeps shackelfordi*. The shelf between the mastoid process and paroccipital process is laterally wide, but lacks a smooth, curved trough that has been noted for early carnivoramorphans such as *Oodectes herpestoides* [7] (Character 33, state 1). There is no indication of an extensive attachment area for the entotympanic posterior to the petrosal that would suggest pronounced posterior inflation of entotympanic (Character 36, state 0). The right and left occipital condyles are as distinct as in extant canids, and in SDSNH 107465, each condyle measures approximately 11 mm along its long axis.

#### Mandible

The moderately-deep dentary (Fig. 2C, D) has a gently arching ventral border and gradually-tapering anterior end. The mandibular symphysis of UCMP 170713 is relatively smooth. The location of the anterior mental foramen varies from below the posterior end of p1 in UCMP 85202 to between p1 and p2 in UCMP 170713. Likewise, the posterior mental foramen is located below the posterior border of p2 in UCMP 85202, but between p2 and p3 in UCMP 170713. The anteroposteriorly-expansive coronoid process rises steeply behind m3, and attains the maximum height along its posterodorsal border. The deep masseteric fossa is anteriorly delineated by a well-developed coronoid crest. The mandibular condyle is cylindrical and medially rather robust, but gradually flattens toward the lateral end. The dentary bears a long and dorsoventrally flat angular process that extends as far posteriorly as the mandibular condyle.

#### Dentition

**Figure 4:**
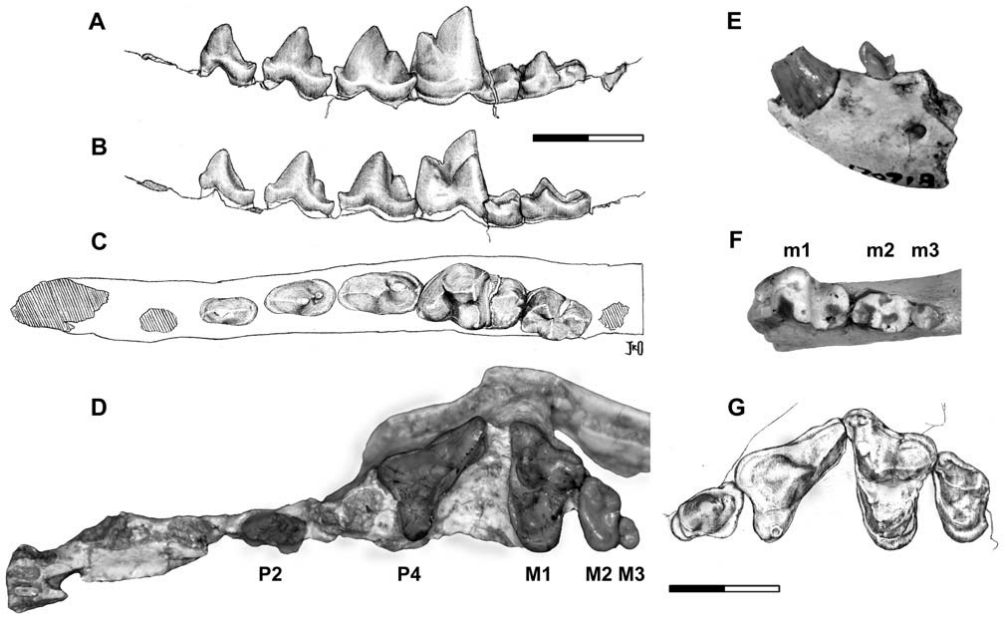
Dentition of *Lycophocyon hutchisoni*. Holotype UCMP 85202 (**A–C**, **G**), UCMP 170713 (**D**, **E**), and SDSNH 107658 (**F**), showing left p2-m2 in labial (**A**), lingual (**B**, inverted), and occlusal (**C**, inverted) views, right P2, P4-M3 in occlusal view (**D**, inverted), left c1 and p1 in labial view (**E**), right m1-m3 in occlusal view (**F**), and left P3-M2 in occlusal view (**G**). Scale bars equal 1 cm.

The dental formula for *Lycophocyon hutchisoni* is 3.1.4.3/?.1.4.3 (Characters 78, 79, 84, 88, state 0). The P1 and lower incisors are not preserved in any of the known specimens. Overall, the dentition of *L. hutchisoni* (Fig. 4) is characterized by: (1) a posterior accessory cusp on P3; (2) well-developed, somewhat blade-like posterior accessory cuspsid on p3 and p4; (3) M1 with a labially extended parastylar region, a protocone with the base that is nearly or partially in contact with the anterolingual margin of the tooth, and an anteroposteriorly asymmetrical lingual cingulum; (4) reduced M2/m2 and diminutive M3/m3 (Character 86, state 1); and (5) m1 and m2 with relatively open trigonids compared to those of earlier carnivoramorphans but without notable reduction (as in *Tapocyon* and feliform carnivorans) or expansion (as in more derived caniform carnivorans) of the talonid.

The upper incisors and canines are preserved in SDSNH 107442. Because of the anterior constriction of the rostrum, the upper incisors (especially I1 and I2) are tightly appressed. The I1 and I2 are subequal in size, mediolaterally compressed as in *Martes pennanti*, and have somewhat spatulate crowns. The I3 is markedly larger than I1 and I2; its crown shows a slight posterior bulging at the base, has a sharp ridge running along its length on the posterolabial side, and is somewhat caniniform in overall morphology. The C1 is of moderate size, and is slightly larger in anteroposterior length than c1.

The P1, P2, and P3 are each preceded by a small diastema (Fig. 4D). Based on the alveolus of SDSNH 107659, P1 appears to have been single-rooted and shorter than P2. The size of upper premolars gradually increases from P1 to P4. The double-rooted P2 of UCMP 170713 (Fig. 4D) is mediolaterally compressed and has a simple triangular profile, with the main cusp showing slight posterior inclination. The tooth lacks a clearly-defined anterior cingulum, but has a small, blade-like posterior accessory cusp that is aligned with the posterior ridge of the main cusp. The posterior accessory cusp is flanked by two small notches, and is followed by a trenchant ridge on the moderately-broad posterior cingulum.

The P3 (Fig. 4G) is labiolingually robust, and has a more asymmetrical profile than P2 because of the better-developed posterior accessory cusp (Character 58, state 0). As in P2, the posterior accessory cusp of P3 is surrounded by a pair of small notches, but the accessory cusp itself is slightly more conical. The anterior cingulum is weakly-developed as a small bulge at the anterior base of the main cusp. In UCMP 85202, the base of the crown bulges out lingually behind the main cusp, but this bulging is less conspicuous in SDSNH 107659. No lingual cusp is present on P3 (Character 80, state 0).

The protocone of P4 (Fig. 4D, G) is located anterior to the paracone (Character 82, state 0); it has approximately one-third of the height of the paracone, and is comparable in size to those of *Daphoenus* and *Amphicticeps shackelfordi* but not as reduced as in *Hesperocyon gregarius* (Character 56, state 1). The parastyle is a diminutive swelling located at the base of the well-defined preparacrista (Character 55, state 2), and is contiguous with the anterior cingulum. The prominent paracone is more posteriorly inclined than the main cusps of P2 and P3, and bears a postparacrista that is nearly as long as the metasylar blade. The sharp metastylar blade is separated from the postparacrista by a deep carnassials notch, and forms a large surface for shearing against the anterior surface of m1 (Character 81, state 0; Characters 54 and 57, state 1). In UCMP 170713 and the right P4 of UCMP 85202, the posterior end of the metasylar blade is labially deflected, but this is less apparent in SDSNH 107659 and the left P4 of UCMP 85202, and appears to reflect variation in individual tooth development. The lingual shearing surface consisting of the postparacrista and the metasylar blade forms an angle of approximately 45° with the long axis of upper tooth row. In UCMP 170713 and UCMP 85202, the cingulum is well delineated around the tooth except at the base of the protocone, and is particularly well-developed at the lingual base of the metasylar blade, contributing to the somewhat inflated appearance of this region in occlusal view. In SDSNH 107659, the cingulum on the posterolingual surface is limited to the base of metastylar blade. No hypocone is present on the P4 (Character 83, state 0).

The unreduced M1 (Fig. 4D, G; Character 46, state 0) is marked by the anterolabially elongate parastylar region (Character 44, state 1) that bears a trenchant preparacrista and a parastylar blade extending straight in the labial direction (Character 45, state 1). The parastylar region of *Lycophocyon hutchisoni*, however, is not as labially elongate as in *Tapocyon robustus* and *Procynodictis vulpiceps*, and the parastylar shelf appears relatively broad (Character 51, state 1). In UCMP 85202 and SDSNH 107659, substantial tooth wear is observed along the anterior surface of preparacrista and parastylar blade, as well as along the anterior lingual cingulum. The paracone is noticeably taller than the metacone (Character 48, state 1), but the two cusps are subequal in anteroposterior length. The apices of the paracone and the metacone are connected by a trenchant ridge consisting of the postparacrista and premetacrista. In UCMP 85202 and UCMP 170713, the paraconule is well developed and is considerably larger than the metaconule (Character 49, state 0), the latter of which is present as a somewhat angular projection at the posterolabial corner of trigon basin. The paraconule is separated from the protocone by a notch. The labial cingulum is well developed and forms a relatively thick ridge along the labial margin of the broad stylar shelf, giving the latter a somewhat basined appearance. The height of the protocone is shorter than that of the paracone but is subequal to that of the metacone (Character 42, state 0). The lingual cingulum is continuous around the protocone in UCMP 85202 (Character 41, state 1). In UCMP 170713 and SDSNH 107659, however, the base of protocone is partly confluent with the anterolingual margin of the tooth, thus interrupting the continuity of lingual cingulum around the protocone. In all specimens, the anterior portion of lingual cingulum is a narrow strip, and the posterior portion forms a crescentic shelf that bulges posterolingually, resulting in the characteristically asymmetrical appearance of the lingual portion of the tooth (Character 47, state 1). The development of posterior lingual cingulum is less pronounced than in early caniform carnivorans such as *Daphoenus* and *Hesperocyon*, and whether to identify this structure as a “hypocone” (Character 50; coded as state 2) is a matter of subjective judgment; however, the edge of posterior lingual cingulum in SDSNH 107659 is slightly worn, suggesting its contact with the anterior portion of the m2 trigonid and involvement in mastication.

The parastylar region of M2 (Fig. 4D, G) projects labially and bears a broad stylar shelf posterior to the parastyle. The short parastyle extends anterolabially until it reaches the anterior margin of tooth, and is separated from the preparacrista by a small notch. A diminutive caspule is present on the anterolabial margin of the stylar shelf and labial to the parastyle. The labial margin of the stylar shelf forms a raised ridge as in M1. The paracone is slightly taller and longer than the metacone. The notched ridge formed by the postparacrista and premetacrista is less trenchant than in M1. In UCMP 85202 and SDSNH 107659, a narrow wear facet is present along the margin of tooth anterior to the paracone. The broad trigon basin is mostly flat because of the diminutive size of paraconule and the absence of metaconule. The protocone is a low, round ridge that is anteriorly more or less confluent with the broad, bulbous lingual cingulum. No hypocone is present on the M2 (Character 87, state 0). Considerable variation in the size of M2 (Character 52; coded as state 0 to be consistent with the coding for other carnivoramorphans in [7]) exists among known specimens: the M2 of UCMP 85202, for example, is approximately 29% longer and 22% wider than that of UCMP 170713. Likewise, the size of M2 (measured as the product of anteroposterior length and transverse width) relative to that of M1 ranges from approximately 0.33 in UCMP 170713 to 0.44 in UCMP 85202.

The diminutive M3 (Fig. 4D; Character 53, state 0) is preserved only in UCMP 170713. It has an oval outline in occlusal view, and a slightly concave anterior margin that closely fits the convex posterolingual margin of M2. The round trigon basin is bordered anteriorly by a slightly crenulated ridge, and labially by two small ridges that may represent reduced paracone and metacone. The single root of the tooth is attached to a groove at the posterior extremity of maxilla along the upper tooth row, such that its posterior surface is not in contact with any bone. This does not seem to be a result of breakage, since none of the known maxillae of *Lycophocyon hutchisoni* has an M3 alveolus that is completely enclosed by the bone. In UCMP 85202, for instance, a posteriorly-exposed M3 alveolus is present at the apparent posterior end of maxilla.

The crown of c1 (Figs. 2D, 4E) at its base is slightly bulbous and anteriorly inclined. In SDSNH 107442, the crown curves rather abruptly at mid-length, such that its tip is oriented more or less vertically. The c1 is labiolingually compressed and has an oval cross section.

All lower premolars (Fig. 4A–C, E) are mediolaterally compressed and bear a well-developed posterior cingulid. Well-defined central ridges are present on the anterior and posterior slopes of the crowns. The size of crown increases gradually from p1 to p4. The single-rooted p1 of UCMP 170713 (Fig. 4E) has an anteriorly-projecting main cuspid and lacks an anterior cingulid. The sharp, highly-tilted posterior ridge of the main cuspid is connected to an anteroposterior ridge on the posterior cingulid that divides the cingulid into a relatively flat, broad lingual portion and a more inclined, narrow labial portion. A pointed cuspulid is located at the posterior end of this ridge on the cingulid.

The double-rooted p2 (Fig. 4A–C) has a main cuspid that rises vertically. A small bulge on the anterolingual margin of the main cuspid forms the poorly-defined anterior cingulid. The trenchant posterior ridge of the main cuspid is followed by a longitudinal ridge on the broad posterior cingulid. As in p1, the posterior cingulid is flatter and broader lingual to this ridge.

The p3 (Fig. 4A–C) has a short anterior cingulid with a diminutive cuspulid that is connected to the anterior ridge of the main cuspid. The sharp posterior ridge of the main cuspid is succeeded by a notch and a posterior accessory cuspid. The posterior accessory cuspid is roughly conical in occlusal view, and is located slightly more labially than the main cuspid. Like the main cuspid, the posterior accessory cuspid bears a ridge along its length, which is followed by a short ridge on the posterior cingulid. The transverse asymmetry of the posterior cingulid across this ridge is more pronounced than in p1 and p2. In occlusal view, the posterior portion of the tooth appears inflated relative to its anterior portion because of the broad posterior cingulid.

The p4 (Fig. 4A–C) has the same basic form as the smaller p3, but is distinguished by a better-developed, trenchant cuspulid on the anterior cingulid, and a longer, more blade-like posterior accessory cuspid. A deep notch is present both anterior and posterior to the posterior accessory cuspid. In labial view, the posterior cingulid ascends posteriorly, and is therefore more elevated than in p3. The posterolingual surface of the main cuspid and the lingual surface of the posterior accessory cuspid form a slight concavity to accommodate the protocone of P4.

The m1 and m2 (Fig. 4A–C, F) are both characterized by a trigonid with relatively robust cuspids and the angle between the paralophid and the protolophid (approximately 65° in UCMP 85202) that is intermediate between those of earlier carnivoramorphans such as *Miacis parvivorus* (with closed trigonid) and early crown-group carnivorans such as *Hesperocyon* (with open trigonid). The trigonid of m1 is roughly 80% longer than the talonid. In contrast to early canids, the metaconid of m1 is unreduced and has nearly the same height as the paraconid. The angle between the paralophid and the line connecting the apices of paraconid and metaconid is approximately 44°. A deep notch is present between the paraconid and the protoconid, and between the protoconid and the metaconid. A deep, wedge-shaped cleft is present between the paraconid and the metaconid. The talonid basin (Character 85, state 0) is relatively narrow but moderately deep, and is demarcated by a continuous ridge, in which the sharp cristid obliqua runs roughly parallel to the paralophid. Vestigial cuspids and cuspulids give a crenulated appearance to this ridge encircling the talonid basin: While the pointed hypoconid is readily recognizable, the rather tightly-appressed entoconid and hypoconulid are diminutive, and are flanked by a distinct bulge on the labial side and two small cuspulids on the lingual side. In UCMP 170713, the entoconid and hypoconulid are barely discernible, and the accessory cuspulids are essentially absent. The labial surface of talonid descends less steeply than in *H. gregarius* to meet the posterior labial cingulid near the base of the crown. The well-defined anterior labial cingulid forms a thin strip.

The trigonid and talonid of m2 (Fig. 4A–C, F) are subequal in length and, together with the well-developed anterior labial cingulid, give the tooth a nearly rectangular outline in occlusal view (Character 59, state 1). The trigonid is considerably more closed than in m1. The trigonid cuspids are low in height but retain pointed apices. The protoconid and metaconid are subequal in height and slightly taller than the paraconid, which has approximately the same height as the hypoconid and is not as markedly reduced as in early canids. A small notch separates each pair of trigonid cuspids. In UCMP 85202, wear facets are present along the posterior cingulum of M1 and the anterior cingulum of M2, indicating shearing against the paralophid and the protolophid of m2, respectively. The talonid is similar in shape to that of m1, but the basin is shallow, in part because the hypoconid is short. The hypoconulid and entoconid are not recognizable as individual structures.

The single-rooted m3 of SDSNH 107658 (Fig. 4F) is low-crowned and is oval in occlusal view. The crown morphology is obscured by heavy wear, but the unworn portions are suggestive of a simple, button-like crown with no clear distinction between the trigonid and the talonid. Comparison with UCMP 170713 suggests that the tooth occluded mostly with M3, with little contact with M2.

In comparison to other North American carnivoramorphans, the dental morphology of *Lycophocyon hutchisoni* appears most similar to those of *Procynodictis vulpiceps*, *P. progressus*, “*Miacis*” *gracilis* (considered by some authors to be synonymous with *P. vulpiceps* [50], [51]), and *Prohesperocyon wilsoni* (morphologically the most-primitive, but not the earliest-known, stem canid [50]). Of these, the first three species are known from the Uintan NALMA, while *P. wilsoni* is known from the Chadronian NALMA. Interestingly, however, even greater resemblance is observed with specimens of *Cynodictis lacustris* from the late Eocene of France. Comparisons with UCMP 62709 and UCMP 63054 from La Débruge, Vaucluse, and AMNH FM 10056 (in collection of the American Museum of Natural History, New York, New York, U.S.A.; identified as *C. intermedius*, which may be conspecific with *C. lacustris* [52]) from a locality of Phosphorites du Quercy in Escamps, Lot, reveals striking similarities in the size and structure of lower premolars (with weakly-developed cuspulids on cingulids and well-developed posterior accessory cuspids on p3 and p4), lower molars (with similar, intermediate openness of m1 trigonid and reduction of m2), and the dentary (including the locations of mental foramina and diastema). The only major differences between the two genera are the better-developed (though still small) entoconid of m1, which makes the posterolingual corner of talonid appear more orthogonal, and the somewhat more elongate talonid of m2 in *C. lacustris*.

As for the upper dentition, UCMP 63173, an isolated P4 of *Cynodictis* sp. from Escamps, is essentially indistinguishable from that of UCMP 170713. An isolated M1 (UCMP 63175) from the same locality also closely resembles that of *Lycophocyon hutchisoni* in the configuration and development of cusps and cingulae, although the labial extension of parastylar region is less pronounced and the posterior lingual cingulum is enlarged in the specimen from France. In addition, the presence of a posterior accessory cusp on P3 (also present in *L. hutchisoni*, *Daphoenus*, and early canids) can be confirmed for a specimen of *Cynodictis* sp. from Quercy (cf. [45]:fig. 8). The phylogenetic affinity of *Cynodictis* to amphicyonids (and, in early studies, canids) has been suggested based on the dental [4], [45], [53] and basicranial morphological similarities [44], [45], [54]. Hunt [54] considered *Cynodictis* to be the earliest known genus of amphicyonine amphicyonids, a Eurasian lineage that is distinct from the North American daphoenine amphicyonids.

#### Caudal vertebra

**Figure 5:**
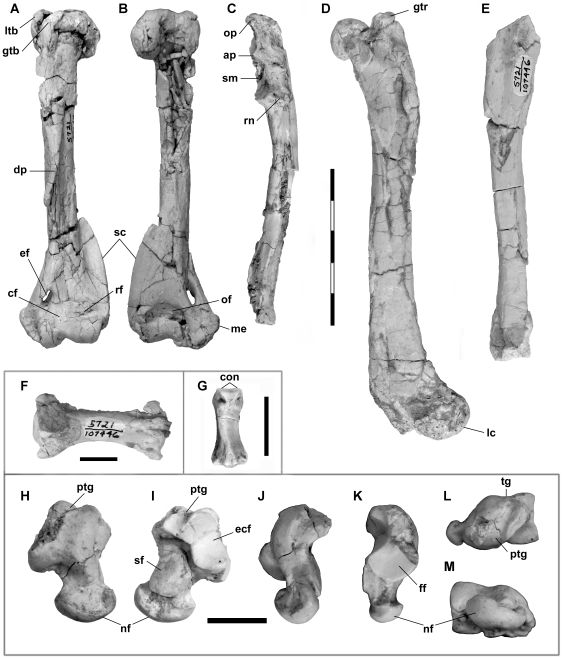
Postcrania of *Lycophocyon hutchisoni*. SDSNH 107447 (**A–B**) and SDSNH 107446 (**C–M**), showing left humerus in anterior (**A**) and posterior (**B**) views, left ulna in lateral view (**C**), left femur in lateral view (**D**), right tibia in medial view (**E**), caudal vertebra in dorsal view (**F**; proximal end to the left), middle phalanx in dorsal view (**G**; distal end to the top), and right astragalus in dorsal (**H**), ventral (**I**), medial (**J**), lateral (**K**), proximal (**L**), and distal (**M**) views. **Abbreviations**: **ap**, anconeal process; **cf**, coronoid fossa; **con**, phalangeal condyles; **dp**, deltopectoral crest; **ecf**, ectal facet; **ef**, entepicondylar foramen; **ff**, fibular facet; **gtb**, greater tuberosity; **gtr**, greater trochanter; **lc**, lateral condyle; **ltb**, lesser tuberosity; **me**, medial epicondyle; **nf**, navicular facet; **of**, olecranon fossa; **op**, olecranon process; **ptg**, plantar tendinal groove; **rf**, radial fossa; **rn**, radial notch; **sc**, supinator crest; **sf**, sustentacular facet; **sm**, semilunar notch; **tg**, trochlear groove. Scale bar equals 5 cm for **A–E**, 1 cm for **F–M**.

Based on the size of transverse processes and the apparent lack of zygapophyses, the caudal vertebra of SDSNH 107446 (Fig. 5F) appears to belong to the proximal portion of the distal caudal vertebral series, but the poor preservation of processes precludes definitive identification. The vertebra is similar to the 8th caudal vertebra of *Nasua narica* (white-nosed coati) in overall size and the development of proximal processes. Its robusticity index of 23 (calculated as the percent proportion of the transverse width of the centrum at its mid-length to the length of the vertebra [55]) is comparable to those obtained for the 8th caudal vertebrae of *Nasua*, *Procyon*, and *Genetta* (genets), and is suggestive of a long, relatively robust tail [55].

#### Humerus

The left humerus of SDSNH 107447 (Fig. 5A, B) shows deformation along the proximal one-third of its length due to compression, and the proximoposterior part of diaphysis is shattered. The total length of the humerus (10.5 cm) is comparable to those of *Procyon lotor* and *Urocyon cinereoargenteus* among extant carnivorans. Compared to other Paleogene carnivoramorphans, it is roughly 40% shorter than those of *Daphoenus vetus* (18.5 cm in CM 492 [56] in collection of the Carnegie Museum of Natural History, Pittsburgh, Pennsylvania, U.S.A.) and *Tapocyon robustus* (17.1 cm in SDSNH 36000), nearly identical to that of “*Miacis*” *uintensis* (10.2 cm in AMNH FM 1964 [57]), and 40–50% longer than those of “*Miacis*” *gracilis* (7.6 cm in CM 11900 [58]) and *Hesperocyon gregarius* (7.1 cm in UCMP 126095).

The greater and the lesser tuberosities have roughly the same height as the humeral head. Due to the crushing, however, the precise orientations of these tuberosities, as well as the form of the humeral head cannot be determined. The morphology of the intertubercular groove is likewise obscured, but it appears to have been well defined. The deltoid and the pectoral ridges converge near the mid-shaft and extend further distally as a prominent deltopectoral crest similar to those in other Paleogene carnivoramorphans such as *Vulpavus* [59], “*Miacis*” *uintensis* [57], and *Tapocyon robustus*. In comparison, the deltopectoral crests of *Hesperocyon gregarius* and all the extant carnivorans examined are much less developed, generally forming a low ridge rather than a flange.

The supinator crest forms a large flange that merges proximally with the diaphysis at approximately 40% of the length of humerus from its distal end, and is comparable to that of *Nasua narica* in this regard. A similarly well-developed supinator crest is present in *Tapocyon robustus* and *Daphoenus vetus* (cf. [1]:plate 20, fig. 15); the same crests in “*Miacis*” *gracilis* [58] and *Hesperocyon gregarius* are much less prominent. The medial epicondyle is well developed and has a rugose surface. A large, elliptical entepicondylar foramen is present proximal to the trochlea. The trochlea, which may be slightly bent due to compression, is approximately half as wide as the capitulum, nearly semicircular in medial view, and projects slightly more distally than the capitulum. The capitulum is rather bulbous and shows slight proximodistal constriction toward its medial end, where it merges with the trochlea. Both the trochlea and the capitulum are relatively shallow in the anteroposterior direction. On the posterior side of the distal humerus, a deep, groove-like depression is present between the medial epicondyle and the trochlea, probably representing the attachment site for the ulnar collateral ligament [59]. The olecranon fossa is well defined but notably shallow as in *Nasua narica*, *Potos flavus* (kinkajou), *Arctictis binturong* (binturong), and apparently *Uintacyon* (cf. [59]:text-fig. 2C). This is in contrast to the deep fossae in “*M.*” *gracilis*, *Daphoenus vetus* (cf. [56]:plate 19, fig. 7), *H. gregarius* (supratrochlear foramen is present in UCMP 126095), and reportedly “*M.*” *uintensis* [57], as well as the extant terrestrial, semi-fossorial, and some of the scansorial carnivorans examined. The coronoid fossa immediately proximal to the trochlea on the anterior side is shallow but well delineated as in *Uintacyon* [59]. A similarly shallow radial fossa is present lateral to the coronoid fossa and proximal to the capitulum.

#### Ulna

The left ulna of SDSNH 107446 (Fig. 5C) is missing the distal end, and the proximolateral surface of the olecranon process is abraded. The olecranon process is relatively straight and does not project any more anteriorly than the anconeal process (the latter, however, may be broken in the specimen). The morphology of the tendinal groove is mostly unrecognizable due to the abrasion, but a flat surface of the proximomedial end of olecranon process suggests the presence of a shallow groove. The semilunar notch appears to have a greater radius of curvature than that in any of the carnivoramorphans examined and “*Miacis*” *uintensis* (cf. [57]:fig. 1), but is comparable to that of *Vulpavus* [59]. A thin stretch of shallow depression is present on the medial surface distal to the semilunar notch, likely representing the insertion site for the antebrachial flexor muscles brachialis and clavobrachialis [60]. The morphology of the radial notch may be slightly obscured by crushing, but it appears to have been relatively wide and flat as in most of the extant mustelids and viverrids examined.

The diaphysis is mediolaterally narrow, and its anterior surface flattens toward the distal end, giving rise to a well-developed, medially-projecting flange for the insertion of the pronator quadratus muscle [60]. Shallow grooves run on the medial and the lateral sides of diaphysis along its length, delineating the sites of attachment for the flexor and extensor muscles of the manus and manual digits [60].

#### Femur

The left femur of SDSNH 107446 (Fig. 5D) exhibits anteroposterior and mediolateral crushing along the proximal and the distal halves, respectively. It is missing most of the medial condyle, and the lesser trochanter is broken. The length of the femur (13.4 cm) is nearly identical to that reported for “*Miacis*” *uintensis* [57] and approximately 50% longer than that of “*M.*” *gracilis* [58]. The shape of the patellar groove is obscured by the crushing. The femoral neck is rather short as in *Potos flavus* and “*M.*” *gracilis* (cf. [58]:fig. 2), and the greater trochanter projects only as far proximally as the femoral head. The presence of the third trochanter cannot be determined due to poor preservation.

#### Tibia

The right tibia of SDSNH 107446 (Fig. 5E) is mediolaterally crushed and is missing both the proximal and distal epiphyses. The diaphysis is mediolaterally narrower than is anteroposteriorly deep, and is intermediate in robusticity between those of *Nasua narica* and *Arctictis binturong*. The prominent ridges on the posterior and posterolateral surfaces of the diaphysis are suggestive of a strong flexor longus hallucis muscle for the flexion of pedal digits [61].

#### Astragalus

The right astragalus of SDSNH 107446 (Fig. 5H–M) is missing a portion of the dorsolateral margin and the proximomedial end of trochlea due to breakage, but is otherwise well preserved. The overall size of the astragalus is similar to that of “*Miacis*” *uintensis* [57].

The medial portion of the trochlea bears a round, very low ridge that smoothly merges into the shallow trochlear groove and the gently-sloping medial side of trochlea (Fig. 5H). The lateral portion of the trochlea, on the other hand, forms a sharp ridge, with a slightly concave fibular facet on its lateral side. In these features, the astragalus of *Lycophocyon hutchisoni* is similar to those of *Martes pennanti*, *Gulo gulo* (wolverine), and *Ailurus fulgens* (red panda). A broad and shallow astragalar trochlea has also been reported for “*Miacis*” *uintensis* [57]; the trochlear groove in *Daphoenus vetus*, however, is noticeably deeper (cf. [1]:plate 20, fig. 22). The dorsal excursion of the plantar tendinal groove (Fig. 5I, L) indicates that the lateral aspect of the trochlear groove does not extend as far proximally as the medial aspect, and the lateral margin of the trochlea has a markedly smaller radius of curvature than the medial margin of the trochlea (Fig. 5J, K). Unlike in basal carnivoramorphans such as *Didymictis*, *Miacis*, *Uintacyon*, and *Vulpavus*, the astragalus appears to lack both the dorsal and ventral astragalar foramina [59], [62], [63]. A relatively long, narrow, and deep plantar tendinal groove for the tendons of the plantarflexor muscles is present proximal to the lateral aspect of the trochlear groove, and extends to the ventral side of astragalus. This groove is oriented slightly oblique to the trochlear groove as in *Vulpavus* and *Didymictis* [59]. Similar to *Vulpavus* and unlike *Didymictis* [59], the astragalus lacks the cotylar fossa.

The sustentacular facet is mediolaterally wide and relatively flat (Fig. 5I), resembling those of *Vulpavus* [59] and, among extant carnivorans, *Nasua narica*. The ectal facet is similar to those of *N. narica* and *Ailurus fulgens* in the shape of its outline and the concave curvature; it also resembles those of *A. fulgens* and *Paradoxurus hermaphroditus* (Asian palm civet) in the slightly helical arrangement of its proximal and the dorsal aspects. The sustentacular and the ectal facets are separated by a deep, narrow depression, and their relative sizes and positions are quite similar to those of *N. narica*. In addition, the outline shapes and relative sizes of these facets are generally similar to those of “*Miacis*” *uintensis* (cf. [57]:fig. 2).

In the distal view (Fig. 5M), the astragalar head is dorsoventrally shallower than in any of the extant carnivorans examined, and its long axis is more or less parallel to the transverse axis of trochlea as in *Vulpavus* [59], *Hesperocyon gregarius* [64], and the extant procyonids and mustelids examined, but in contrast to the markedly more tilted astragalar heads in *Didymictis* [59], “*Miacis*” *gracilis*, *Atilax paludinosus* (marsh mongoose), and extant canids [64]. The dorsoventral and mediolateral convexity of the navicular facet is comparable to those in *Nasua narica* and *Procyon lotor*.

#### Phalanx

The middle phalanx of SDSNH 107446 (Fig. 5G) is characterized by the asymmetrical diaphysis with one side of the dorsal aspect forming a much broader slope than the other. This phalanx cannot be sided on the basis of the asymmetry because the sloping dorsal aspect may face medially or laterally in extant carnivorans, depending on the taxon and the digit. The asymmetry is not associated with deep excavation of the diaphysis or lateral protrusion of the articular condyle as seen in extant felids and, to a lesser degree, in *Tapocyon robustus* (cf. [49]:fig. 7), in which these features enable full retraction of the claws [65]. In dorsal view, the outline of phalanx as a whole is essentially symmetrical. Overall, these conditions are similar to those found in some digits of *Daphoenus vetus* (cf. [1]:plate 20, fig. 21), *Ailurus fulgens* and extant canids such as *Vulpes vulpes* (red fox) and *Urocyon cinereoargenteus*.

### Assessment of Size Variation for Taxonomic Consideration

**Figure 6:**
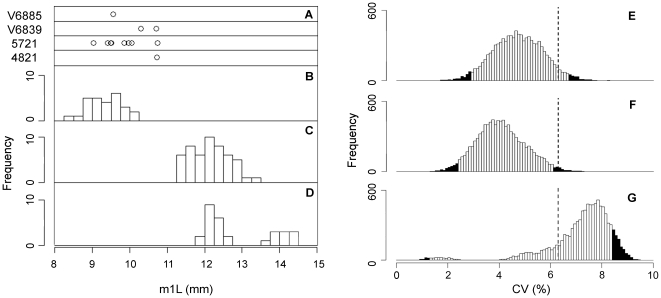
Comparisons of within-sample variation in m1L of *Lycophocyon hutchisoni* and selected carnivorans. Measurements of *L. hutchisoni* from UCMP localities V6839 ( = RV6830) and V6885, and SDSNH localities 4821 and 5721 (**A**) are plotted on the same scale as the histograms for samples of *Hesperocyon gregarius* (**B**), *Urocyon cinereoargenteus townsendi* (**C**), and *Martes pennanti columbiana* (**D**). Histograms of CV values for 10,000 bootstrapped pseudo-replicates (each consisting of 9 specimens) of *H. gregarius* (**E**), *U. c. townsendi* (**F**), and *M. p. columbiana* (**G**) are compared to observed CV for 9 specimens of *L. hutchisoni* (6.3%; dashed lines); bootstrap-based CV values that fall outside the 95% confidence intervals are shaded in black.

Specimens of *Lycophocyon hutchisoni* exhibit notable size variation (Fig. 6A). For example, the anteroposterior length of the lower first molar (m1L) ranges from 9.1 mm in SDSNH 107450 to 10.8 mm in SDSNH 107458, representing a difference of 19%. Because size difference is sometimes the only observable distinction between closely-related species of fossil mammals [66], [67], the possibility that the known specimens of *L. hutchisoni* in fact represent more than one species was evaluated by comparing the coefficient of variation (CV) [68] in m1L to those of the earliest known stem canid, *Hesperocyon gregarius*, an extant canid, *Urocyon cinereoargenteus* (gray fox), and an extant mustelid, *Martes pennanti* (fisher). These comparative taxa were selected based on their sizes (Fig. 6B–D) and m1 morphology (with well-developed carnassial shear and talonid) that are reasonably similar to those of *L. hutchisoni*. In addition, *H. gregarius* represents a fossil species with an adequate sample size that is phylogenetically close to *L. hutchisoni* (see the result of a cladistic analysis presented below); *U. cinereoargenteus* and *M. pennanti* represent species with low and high degrees of sexual size dimorphism, respectively (Fig. 6D). Since diagnosis of fossil taxa is prone to subjective lumping or splitting of morphotypes by researchers, the comparison with another fossil species, *H. gregarius*, may appear circular for the purpose of recognizing the species boundary of *L. hutchisoni*. However, the morphological integrity both in size and form of *H. gregarius* (i.e., the species is not clearly divisible into smaller sets of morphotypes) has been well established by comparison to extant species of canids [50]. As may well be the case for the sample of *L. hutchisoni*, the sample of *H. gregarius* consists of geologically-diachronous individuals, providing a useful reference for exploring possible accumulation of size variation over the history of an evolutionary lineage segment. At the least, comparison with well-sampled, clearly-delineated fossil species such as *H. gregarius* should contribute to consistency in taxonomic practice by establishing reasonable size ranges for closely-related fossil species. It should also be noted that, because different species in a sample (paleontological or otherwise) need not differ in size, presence of a single species in a sample cannot be demonstrated by statistical hypothesis testing; instead, the purpose of cross-taxonomic comparison here is to inform a taxonomic decision by testing whether the observed within-sample variation of *L. hutchisoni* is too great to be interpreted as solely intraspecific variation.

The sample-size adjusted coefficient of variation (CV) [69] in m1L is 6.3% for 9 specimens belonging to separate individuals of *Lycophocyon hutchisoni*. Statistical hypothesis tests using randomization procedure [70] (see Materials and Methods for details) show that the CV of 6.3% for *L. hutchisoni* falls within the bootstrap estimates of bias-corrected 95% confidence intervals for *Hesperocyon gregarius* (95% CI = [2.9%, 6.7%], mean = 4.7%, median = 4.7%; Fig. 6E) and *Martes pennanti* (95% CI = [1.3%, 8.4%], mean = 7.2%, median = 7.5%; Fig. 6G), but outside that for *Urocyon cinereoargenteus* (95% CI = [2.4%, 6.1%], mean = 4.1%, median = 4.0%; Fig. 6F). Thus, at the confidence level of *α* = 0.05, the within-sample variation in m1L of *L. hutchisoni* is statistically indistinguishable from those of *H. gregarius* and *M. pennanti*, but is significantly greater than that of *U. cinereoargenteus*.

### Dietary Inference

**Figure 7:**
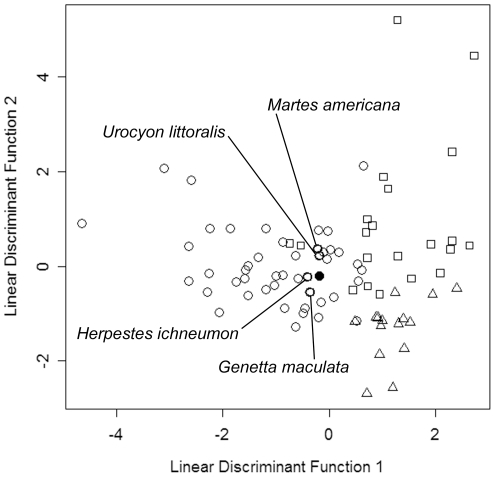
Discriminant function plot of extant carnivorans and *Lycophocyon hutchisoni*. Six ecomorphological variables were used to maximally separate three dietary groups: carnivores (open circles), omnivores/hard-object feeders (open squares), and insectivores (open triangles). Data for 82 extant taxa are from Friscia et al. [71] and those for *L. hutchisoni* (filled circle) are based on holotype UCMP 85202. Four labeled taxa are the closest to *L. hutchisoni* in their posterior probabilities of dietary-group affiliations.

The body weight of the individual represented by the holotype UCMP 85202 was estimated from its m1L to be roughly 6 kg (see Materials and Methods). A linear discriminant analysis of dietary categories using 6 morphological variables and the data set of Friscia et al. [71] predicted a carnivorous diet for *Lycophocyon hutchisoni*, with the posterior probabilities of 83%, 10%, and 7% for carnivory, omnivory/durophagy, and insectivory, respectively (Fig. 7; see Materials and Methods). This prediction reflects the long m1 trigonid and the small m2, features indicating the relative importance of shearing over crushing when compared to extant carnivorans that are not major consumers of vertebrates (Table 3).

**Table 3:** Dental ecomorphological statistics for *Lycophocyon hutchisoni* and comparative extant carnivorans.

Taxon/Dietary group	*N*	*LBW*	*m1BS*	*m2S*	*RBL*	*RUGA*	*UM21*
*Lycophocyon hutchisoni* ^†^ (holotype)	1	8.73	0.074	0.058	0.682	1.068	0.638
Carnivores	46	7.71	0.101	0.052	0.660	0.807	0.327
Omnivores/Hard-object feeders	21	8.26	0.085	0.069	0.573	1.107	0.334
Insectivores	15	7.01	0.074	0.077	0.630	1.025	0.556

Data on extant carnivorans (mean values) from Friscia et al. [71].

**Abbreviations:** ***LBW***, natural log-transformed body weight in grams; ***m1BS***, length of the m1 trigonid relative to the length of dentary (“M1BS” of [71]); ***m2S***, square-root transformed m2 occlusal area relative to the length of dentary (“M2S” of [71]); ***RBL***, length of the m1 trigonid relative to the length of m1; ***RUGA***, square-root transformed occlusal areas of M1 and M2 combined, relative to the length of P4; ***UM21***, square-root transformed occlusal area of M2 relative to that of M1.

### Cladistic Analysis

**Figure 8:**
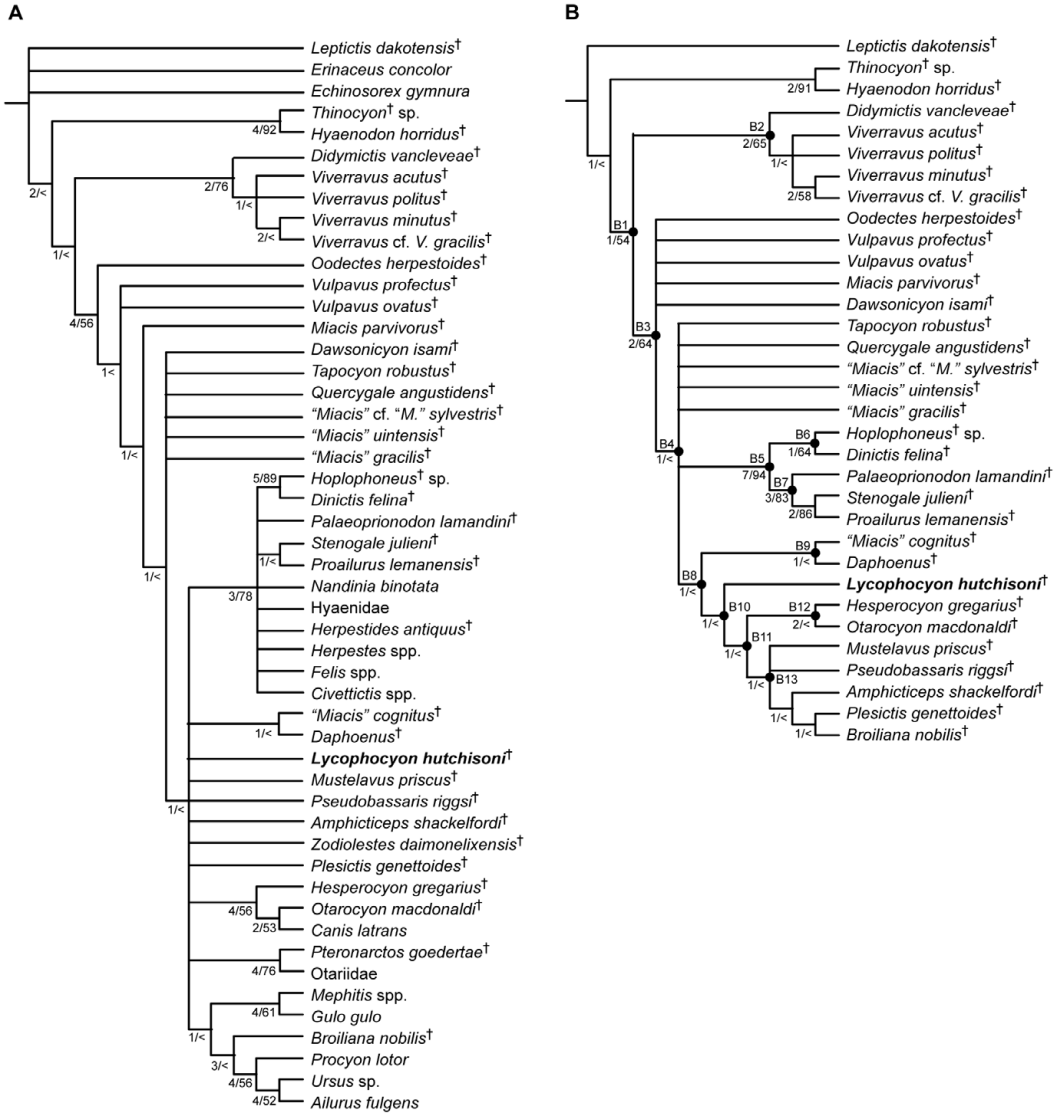
Cladistic position of *Lycophocyon hutchisoni*. **A**, strict consensus of 132 most-parsimonious trees (tree length = 488 steps, ensemble consistency index = 0.289, ensemble retention index = 0.667) obtained for 50 OTUs. **B**, strict consensus of 32 most-parsimonious trees (tree length = 280 steps, ensemble consistency index = 0.382, ensemble retention index = 0.664) obtained for 33 taxa that are known from the Paleogene. Numbers next to branches indicate Bremer support values followed by bootstrap support values. Bootstrap support values below 50% are denoted as “<”.

Building on the currently most-extensive character matrix in the literature for basal carnivoramorphans and early carnivorans [72] (see also [7], [57], [73], [74]), a parsimony analysis of 50 taxa (including 2 hyaenodontid creodonts and 3 outgroup taxa represented by *Leptictis dakotensis*, *Erinaceus concolor*, and *Echinosorex gymnura*) and 98 morphological characters was performed to determine the cladistic position of *Lycophocyon hutchisoni* (see Materials and Methods). This analysis yielded 132 most-parsimonious trees (length = 488 steps, ensemble consistency index = 0.289, ensemble retention index = 0.667; Appendix S3), and failed to resolve the relationships among the carnivoramorphans surrounding the base of crown-group Carnivora, including *L. hutchisoni* (Fig. 8A).

Consequently, a second parsimony analysis was conducted for a subset of the same character matrix consisting of only the taxa that are known from the Paleogene Period (see Discussion) and *Leptictis dakotensis* as the outgroup taxon. The strict consensus of 32 most-parsimonious trees thus obtained (length = 280 steps, ensemble consistency index = 0.382, ensemble retention index = 0.664; Appendix S3) placed *Lycophocyon hutchisoni* on the caniform branch within the Carnivora, and immediately outside the crown-group Canoidea (Fig. 8B). Addition of *Nandinia binotata*, which is consistently identified by molecular studies as belonging to the earliest-splitting lineage among extant feloids [12], [21], [75], [76], does not alter the relationships of other taxa in the strict consensus tree; when included in the cladistic analysis, *N. binotata* is positioned as the sister taxon to the monophyletic group B7 (Fig. 8B). Likewise, the selection of *Thinocyon* sp. or *Hyaenodon horridus* (“*Hyaenodon cruentus*” in [7]) as the outgroup taxon (instead of *Leptictis dakotensis*) does not affect the topology of the consensus tree with respect to the non-viverravid carnivoramorphans.

The topology of the consensus tree for Paleogene taxa broadly agrees with those reported in the recent studies [7], [57], [72]–[74] in that (1) the viverravids form a monophyletic group (B2 in Fig. 8B) outside all other carnivoramorphans, and (2) the earliest non-viverravid carnivoramorphans are located outside the crown-group Carnivora. However, it differs in the ambiguous placement of *Quercygale angustidens*, “*Miacis*” cf. “*M.*” *sylvestris*, “*M.*” *gracilis*, “*M.*” *uintensis*, and *Tapocyon robustus* either inside or outside the crown-group Carnivora. As a result, the precise phylogenetic origin of the crown-group Carnivora cannot be located. In the most-parsimonious trees in which “*M.*” *sylvestris*, “*M.*” *gracilis*, and “*M.*” *uintensis* are included in the Carnivora (18 out of 32 most-parsimonious trees), *Q. angustidens* is invariably positioned as a basal feliform, whereas *T. robustus* is variably located in the Caniformia or the Feliformia (Appendix S3).

*Lycophocyon hutchisoni* shares with the canoids (B11 in Fig. 8B) the derived trait of the broad and flat anterior extension of the petrosal promontorium (Character 28, state 3), but lacks the canoid synapomorphies (though none is unique to the Canoidea) of: (1) the infraorbital foramen positioned above the anterior edge of P4 (Character 4, state 1); (2) loss of M3 (Character 53, state 1); and (3) well-ossified entotympanics firmly fused to the basicranium (Character 68, state 1). The monophyletic group consisting of the canoids, *L. hutchisoni*, “*Miacis*” *cognitus*, and *Daphoenus* (B8 in Fig. 8B) is united by a wide shelf between the mastoid process and the paroccipital process that does not form a trough (Character 33, state 1). Synapomorphies for other selected monophyletic groups in the consensus tree are as follows (see node numbers in Fig. 8B): B1 (Carnivoramorpha), M1 with broad parastylar shelf (Character 51, state 1), carnassials consisting of P4 and m1 (Character 54, state 1), P4 protocone anterior to paracone (Character 82, state 1), pronounced size decrease from m1 to m3 (Character 86, state 1); B2 (Viverravidae), small flange along middle-ear chamber formed by ventral floor of basioccipital (Character 34, state 1), subequal heights of protocone and paracone (Character 42, state 1), absence of m3 (Character 88, state 1); B3, round infraorbital foramen (Character 3, state 1), fenestra cochlea located at the same level or anterior to mastoid tubercle (Character 18, state 1), elongate promontorium with round anterior end (Character 28, state 1), rugose surface for entotympanic attachment on anteromedial promontorium or tympanic wing of basisphenoid (Character 30, state 1), deep fossa for tensor tympani muscle (Character 39, state 1), short m2 talonid (Character 59, state 1); B4, no synapomorphy exists for this clade that is common to all most-parsimonious trees; B5 (part of Feliformia), postglenoid foramen reduced or absent (Character 12, state 1), short promontorium with blunt anterior end (Character 28, state 2), promontorium with facet for ectotympanic attachment (Character 29, state 1), absence of lingual cingulum on M1 (Character 41, state 0; derived among non-viverravid carnivoramorphans but reversal among all taxa considered in the analysis), reduced M1 (Character 46, state 1), pronounced reduction of m1 talonid (Character 85, state 1); B6 (Nimravidae), reduced paroccipital process (Character 9, state 1), mastoid process extending farther than paroccipital process (Character 13, state 0; derived among non-viverravid carnivoramorphans but may represent a reversal within the Carnivoramorpha), M1 with narrow parastylar shelf (Character 51, state 2), well-ossified entotympanic firmly attached to basicranium (Character 68, state 1), absence of p1 (Character 84, state 1); B7 (Feloidea), absence of lacrimal exposure on rostrum (Character 1, state 2), condyloid foramen close to posterior lacerate foramen (Character 15, state 1), extensive attachment area for entotympanic on promontorium posterior to fenestra cochlea (Character 26, state 1), narrow shelf between mastoid process and paroccipital process (Character 33, state 2), middle lacerate foramen located anterior to basisphenoid-basioccipital suture (Character 40, state 2), anterior entry of carotid artery into auditory capsule not enclosed in bony tube (Character 67, state 3); B9, anteriorly open fossa for stapedius muscle (Character 37, state 1); B12 (stem-group Canidae), condyloid foramen close to posterior lacerate foramen (Character 15, state 1), anterior lingual cingulum of M1 reduced or absent (Character 41, state 2), absence of parastylar shelf on M1 (Character 51, state 0; derived within the Carnivoramorpha but a reversal among all taxa considered in the analysis); B13 (Arctoidea), absence of hypocone on M1 (Character 50, state 0; derived within the Canoidea but a reversal within the Carnivoramorpha).

Finally, the tree length increases by at least: (1) 2 steps when *Lycophocyon hutchisoni* is paired with *Daphoenus* or the group consisting of *Daphoenus* and “*Miacis*” *cognitus*; (2) 6 steps when *L. hutchisoni* is placed in the Feliformia; (3) 5 steps when *L. hutchisoni* is placed immediately outside the Carnivora; and (4) 5 steps when the group consisting of *Daphoenus* and “*M.*” *cognitus* is either paired with or placed among the basal arctoids in the analysis.

## Discussion

### Intraspecific Variations in Dental Morphology

While the size variation among known specimens of *Lycophocyon hutchisoni* is notable, it does not significantly exceed that of *Martes pennanti*, an extant mustelid with a high degree of sexual size dimorphism, or that of *Hesperocyon gregarius*, an extinct canid (Fig. 6E, G). From the perspective of hypothesis testing, this should be viewed not as direct support for the presence of a single species in the sample of fossil specimens but as failure to detect the presence of more than one species. A comprehensive assessment of intraspecific size variation in carnivorans was not attempted in the present study, in part to minimize the statistical problem of multiple comparisons; nevertheless, CVs in m1L of other carnivorans available in the literature (Table 4) are consistent with the interpretation that the size variation of *L. hutchisoni* is not unusually high compared to those of extant carnivoran species.

**Table 4:** Coefficients of variation in m1L of selected carnivorans.

Taxon	*N*	Mean (mm)	CV (%)^1^	Source
*Lyocphocyon hutchisoni* ^†^	9	9.88	6.3	This study
**Canidae**				
*Hesperocyon gregarius* ^†^	27	9.35	4.8	This study
*Urocyon cinereoargenteus*	51	12.15	4.1	This study
*Urocyon cinereoargenteus*	81	12.41	5.2	[128]
*Vulpes lagopus*	58	13.80	4.5	[145]
*Vulpes vulpes*	50	15.38	4.2	[67]
**Mustelidae**				
*Martes americana*	121	8.84	7.2	[128]
*Martes pennanti*	29	12.89	7.3	This study
**Felidae**				
*Felis sylvestris*	21	8.18	7.4	[80]

1 Adjusted for sample size [69].

The significantly-greater CV in m1L of *Lycophocyon hutchisoni* compared to that of *Urocyon cinereoargenteus* (Fig. 6F) merits discussion. Because the geographic area encompassed by the sample of *U. cinereoargenteus* is much greater than that of *L. hutchisoni* (approximately 73,000 km^2^ versus 3 km^2^), the difference in CV is not attributable to geographic variation. Instead, it may partly be explained as phyletic variation in size of *L. hutchisoni*. Indeed, Hunt [77] demonstrated that greater time-averaging of fossil samples significantly increased the observed variance in quantitative morphological traits as predicted under the Markovian random-walk model of phenotypic evolution. However, Hunt [77], [78] also showed that, for a variety of organisms and traits (including m1L of mammals), the increase in within-sample variance caused by time-averaging of 10^4^–10^5^ years was typically on the order of 1%. This would result in 0.5 to 4% increase in CV, whereas the observed difference is roughly 50%. Therefore, considering that much of the size variation in *L. hutchisoni* is captured by specimens from the same horizon (SDSNH locality 5721; Fig. 6A), it seems likely that the difference in CV between the samples of *L. hutchisoni* and *U. cinereoargenteus* primarily reflects greater intrapopulational variation of the former independent of time.

With regard to qualitative dental morphology, it is notable that the subtle variations among the specimens of *Lycophocyon hutchisoni*, such as the degree of development of accessory cuspids on lower premolars and the continuity of M1 lingual cingulum around the protocone, are well documented both within and across populations of an extant canid, *Vulpes vulpes* [79]. Indeed, the increasing knowledge of intraspecific dental morphological variation in extant carnivorans [79]–[82] is especially pertinent to the taxonomy of fossil species, and should inform the selection of morphological characters and categorization of character states in future cladistic analyses. In summary, the dental morphological variations among the known specimens of *Lycophocyon* appear insufficient for establishing multiple species within the genus.

### Ecomorphological Interpretations

#### Dentition

In addition to predicting a carnivorous diet for *Lycophocyon hutchisoni*, the linear discriminant analysis of ecomorphology produced similar posterior probabilities of dietary-group affiliations for such extant carnivorans as *Urocyon littoralis* (island fox), *Genetta maculata* (rusty-spotted genet), *Martes americana* (American marten), and *Herpestes ichneumon* (Egyptian mongoose; Fig. 7). While the obvious correlations among the predictor variables must be noted, the coefficients of linear discriminants (see Materials and Methods) indicate that these taxa are united among ecological carnivores by their relatively large m2 occlusal areas and relatively short m1 trigonid lengths. Indeed, as would be expected from such dental morphology, insects and plants may constitute a significant portion of the diet of *U. littoralis*, *G. maculata*, and *M. americana* [83]–[86]. Likewise, *H. ichneumon*, while most heavily dependent on small terrestrial vertebrates (as measured in consumed biomass), often feeds on insects, and its opportunistic diet may also include fish, hard-shelled aquatic invertebrates, and plants [83], [87], [88]. Notable similarity in dietary composition between *G. maculata* and *H. ichneumon* where they are sympatric has been reported [83]. Thus, the observable ecomorphology suggests *L. hutchisoni* to have been a generalist mesocarnivore (sensu Van Valkenburgh [89]).

#### Postcranial skeleton

The locomotor inference for fossil mammals is necessarily based on comparison of their skeletal forms with those of their extant relatives, for which direct behavioral observations are available (cf. [59], [64], [90]). To alleviate the potential problem of allometry, comparisons were made primarily with extant carnivorans of similar body size. Decoupling the phylogenetic and adaptive components of postcranial skeletal morphology is difficult at present, but it is plausible that, in some cases, relatively minor skeletal modifications can provide sufficient adaptations for highly divergent locomotor habits; for example, postcranial elements of the extant *Urocyon cinereoargenteus* (gray fox) are clearly recognizable as belonging to a canid (author's pers. obs.), yet this species, unlike other canids, is highly capable of climbing trees [91]. In light of the recent advancement in molecular phylogenetics of carnivorans, comprehensive studies of their locomotor morphology in explicitly phylogenetic frameworks (cf. [92]) are awaited.

The humeral morphology of *Lycophocyon hutchisoni* (Fig. 5A, B) is suggestive of an adept climber. The mobility of the glenohumeral joint is enhanced by the low height of the greater tuberosity [59], [93]. The distal extension of the prominent deltopectoral crest resembles the condition in *Vulpavus* [59] and arboreal *Nandinia binotata* (African palm civet) [94], and suggests the presence of powerful musculature that generated large force at the expense of speed [95]. The well-developed medial epicondyle and the expansive supinator crest are similar to those of *Nasua narica* (white-nosed coati) and *Gulo gulo* (wolverine), which are both skilled climbers [96]–[98], and are indicative of strong flexor and extensor muscles for the manus and the manual digits that are necessary for habitual climbing [94]. The muscle attachment areas on the humerus of *L. hutchisoni*, however, are not expanded to the same degree as in the semi-fossorial *Taxidea taxus* (American badger). The well-demarcated coronoid fossa similar to that of *N. narica* may reflect frequently flexed position of the ulna, as has been suggested for *Vulpavus* [59]. The very shallow olecranon fossa as seen in *Potos flavus* (kinkajou) and *Arctictis binturong* (binturong) and the limited projection of the humeral trochlea distal to the capitulum suggest a relatively wide range of mediolateral movement of the ulna at the humeroulnar joint, and are in contrast to the typical morphology in extant terrestrial carnivorans that restricts the ulnar movement mostly to the anteroposterior direction [94].

Features of the ulna (Fig. 5C) are consistent with the mode of locomotion inferred from the humeral morphology. The lateral orientation of radial notch enhances the rotation of radius [94]. The relatively straight olecranon process of *Lycophocyon hutchisoni* compared to those of extant terrestrial carnivorans maximizes the force generated by the triceps muscle when the ulna is highly flexed, as often occurs during climbing [90], [94].

With regard to the hind limb, the relatively round head (similar to *Paradoxurus hermaphroditus* (Asian palm civet) and more spherical than in *Urocyon cinereoargenteus* (gray fox) and *Vulpes vulpes* (red fox)) and the short neck of femur (Fig. 5D) suggest its wide range of rotational movement at the hip joint [63].

The shallow trochlear groove and the low, round ridge of the medial trochlear margin of astragalus (Fig. 5H, M) are comparable to those of *Martes pennanti* (fisher), *Gulo gulo*, *Nasua narica*, and *Ailurus fulgens* (red panda), and are suggestive of enhanced pedal inversion concomitant with plantarflexion [59], [96]. The dorsolateral extension of the navicular facet (Fig. 5H, K, M) may have enhanced the eversion of the foot at the astragalonavicular joint [99]. In addition, the ectal facet with a smoothly round concavity and the slightly helical arrangement of its proximal and distal aspects (Fig. 5I) would, together with the mediolateral orientation of the astragalar head (Fig. 5M) and the ventrally-facing sustentacular facet (Fig. 5I), further facilitate the pedal inversion by subtalar joint movement [96]. At the same time, although the proximal extent of the medial trochlear margin (Fig. 5H, J) and the apparent lack of the dorsal astragalar foramen may have allowed the maximum angle between the tibial diaphysis and the long axis of the astragalus to be greater than 90° (cf. [59]), the presence of the dorsally-extensive plantar tendinal groove slightly in angle with the trochlear groove (Fig. 5L) likely limited the range of plantarflexion [64]. The astragalar morphology is thus indicative of a plantigrade posture and substantial hindfoot flexibility, but the ability to completely reverse the hindfoot as in the arboreal *Potos flavus* is unlikely, since it would have required a greater range of plantarflexion [96]. It should be noted, however, that complete reversal of hindfeet is not necessary for descending trees headfirst: the scansorial *N. narica*, for example, is known to compensate for the relatively limited hindfoot flexibility with pronounced abduction of the femora [96].

Taken together, the known postcranial elements of *Lycophocyon hutchisoni* point to a scansorial habit of an animal that was likely as adept at climbing as the extant *Nasua narica* [96], [97] and *Gulo gulo* [98] (although the substantial weight difference between *L. hutchisoni* and *G. gulo* makes the latter comparison less conclusive), but was probably not as dependent on the arboreal habitat as *Potos flavus*.

### Cladistic Position of *Lycophocyon hutchisoni* and Remarks on the Phylogeny of Early Carnivorans

Following recent studies of early carnivoramorphans [7], [57], [72]–[74], with which the present study shares many of the same character matrix data, the initial cladistic analysis here included 12 operational taxonomic units (OTUs) represented partly or entirely by extant carnivorans (Fig. 8A; also see [7]). However, this analysis failed to (1) resolve the cladistic position of *Lycophocyon hutchisoni* and (2) recover well-established relationships of extant arctoids [12], [33], [76], [100]. In re-examining the results of the recent studies [7], [57], [72], [73], it is notable that they consistently reported most-parsimonious trees in which many of the extant (partially or entirely) OTUs clustered together, showing topologies that are in major conflict with those that are strongly supported by recent molecular and combined molecular and morphological studies [12], [33], [76], [100], [101]; interestingly, the same pattern is observed when comparing the morphological tree and the combined morphological and molecular tree of fossil and extant arctoids reported by Finarelli [100]. In the present and previous studies [7], [57], [72], [73], this problem may be attributable to the insufficient sampling of fossil taxa from the Neogene that would fill the morphological gaps between the basal carnivorans and their extant relatives, predisposing the cladistic analysis to long branch attraction (cf. [47], [102], [103]). Temporally-long branches in parsimony analysis are of particular concern in light of the growing evidence that, at least in parts of the carnivoran phylogeny, the types of craniodental characters considered here have evolved more rapidly and flexibly than had traditionally been assumed [22], [101], [104]. Furthermore, the morphological characters analyzed in the present and recent studies [7], [57], [72]–[74] may not be suitable for analyses that include highly-derived extant carnivorans because they were originally selected primarily to resolve the relationships of early carnivoramorphans [7]. In any case, the phylogenetic locus of interest in the present study is not the entire carnivoramorphan tree but the branches surrounding *Lycophocyon hutchisoni*, and so basal carnivorans, rather than extant carnivorans that are morphologically far removed from the carnivoran origin (e.g. see [100] for morphological transformations that separate extant arctoids from their extinct basal relatives), should provide more appropriate and sufficient data for the polarization of character states in this region of the carnivoramorphan tree. For these reasons, the discussion below of basal carnivoran phylogeny is based on the result of the second analysis that focused on the Paleogene carnivoramorphans (Fig. 8B).

The following interpretations of the strict consensus tree for the Paleogene taxa (Fig. 8B) rest on the assumptions that (1) at least *Hesperocyon gregarius* or *Otarocyon macdonaldi* is a caniform, (2) at least one among *Stenogale julieni*, *Proailurus lemanensis*, and *Palaeoprionodon lamandini* is a feliform, and (3) at least one among *Mustelavus priscus*, *Pseudobassaris riggsi*, *Amphicticeps shackelfordi*, *Plesictis genettoides*, and *Broiliana nobilis* is an arctoid. These assumptions are deemed secure in light of detailed studies of their skeletal anatomy and previously-conducted cladistic analyses [7], [47], [50], [57], [72], [73], [100], [105], [106]. As in the recent studies that share many of the same character matrix data [7], [57], [72], [73], the nodal support values for the consensus tree are generally low (Fig. 8B), but such information cannot simply be taken as evidence against particular phylogenetic hypotheses when morphological variations among the taxa of interest are limited, as might be expected for basal branches that have been divergent for a relatively short period of time (cf. [47]). It is hoped that further progress in mammalian molecular phylogenetics and developmental genetics will help formulate probabilistic models of skeletal evolution that can be incorporated into future cladistic analyses of the taxa considered here.

The proximity of *Lycophocyon hutchisoni* to one of the earliest-known amphicyonids, *Daphoenus*, agrees with its notable similarity in dental morphology to another early amphicyonid, *Cynodictis lacustris*. Further testing of the hypothesized cladistic position of *L. hutchisoni* would, therefore, benefit from increased sampling of early amphicyonids (*Cynodictis* could not be incorporated into the present cladistic analysis because of the limited availability of specimens that preserve morphological details of the basicranium).

The placement of *Daphoenus* outside the Canoidea (Fig. 8B, node group B11) corroborates the findings of some of the recent studies [7], [74], and is consistent with its earlier first appearance than those of almost all other caniforms (see below). It also implies that the deep excavation of the lateral margin of basioccipital in amphicyonids and ursids—a trait that is often considered as a potential synapomorphy uniting the two groups [14], [46]—may have evolved convergently. In fact, distribution of this trait among the most basal ursids appears to be poorly known at present (cf. [100]). Furthermore, this phylogenetic arrangement is most parsimonious with regard to loss of M3 and ossification of entotympanics in early caniforms. Because early amphicyonids such as *Daphoenus* possess M3s and lack well-ossified entotympanics firmly fused to the basicranium, their inclusion in the Canoidea (regardless of their precise affiliation with ursids) would require (1) an additional loss (two independent losses within canoids) or a regeneration of M3s and, similarly, (2) an additional instance of entotympanic ossification (two independent ossifications within canoids) or a reversal to unossified (or poorly-ossified) entotympanics. Of these possibilities, multiple losses of M3s within the Canoidea are not implausible, but no basal caniform of unquestionable canoid affinity is currently known that retains M3s. On the other hand, a regeneration of M3s as part of the regular dentition seems yet more improbable considering that almost no such case is known among living and extinct caniforms of unquestionable canoid affiliation, the sole exception being *Otocyon megalotis* (bat-eared fox) [107]; the remarkably well-developed “M3s” of *O. megalotis*, whose diet consists mainly of termites [107], are suggestive of an unusual molar developmental system [108]–[110] and are a questionable comparison to the highly-reduced M3s of early amphicyonids that are morphologically similar to those of the non-canoid caniform *Lycophocyon hutchisoni*.

The feliform affiliation of nimravids (*Dinictis felina* and *Hoplophoneus* sp.) is in accord with the findings of several previous studies [7], [14], [111]. The monophyletic subgroup of the Feliformia consisting of the nimravids, *Stenogale julieni*, *Proailurus lemanensis*, and *Palaeoprionodon lamandini* (Fig. 8B, Group B5) has the highest nodal support values of all the monophyletic groups in the cladogram, and is supported by at least five probable synapomorphies (see Results).

Recent studies [7], [57], [72]–[74] consistently placed *Tapocyon robustus*, *Quercygale angustidens*, “*Miacis*” cf. “*M.*” *sylvestris*, “*M.*” *uintensis*, and “*M.*” *gracilis* outside the crown-group Carnivora, suggesting a phylogenetically-shallow origin of carnivorans consisting mostly of the taxa that have long been recognized as definitive carnivorans. Considered in this light, the equivocal relationships of the above-mentioned taxa to crown-group carnivorans in the most-parsimonious trees obtained here are an important finding of the present study, providing an alternative hypothesis of a phylogenetically-deeper origin of carnivorans. Thus, with regard to the timing of caniform-feliform divergence, the consensus tree of Paleogene taxa (Fig. 8B) suggests two possible minimum divergence dates (see [112] for the protocol for deriving minimum constraints on lineage divergence dates):

1. If “*Miacis*” *sylvestris* is located outside the Carnivora, the conservative minimum divergence date will be 38 million years ago (Ma) based on the first appearance of the amphicyonid *Daphoenus lambei* in the early-Duchesnean Hendry Ranch Member of the Wagon Bed Formation, Wyoming, and assuming that the locality is older than the Buckshot Ignimbrite of Texas, which has yielded a ^40^Ar/^39^Ar date of 37.8±0.2 Ma [53], [113], or based on the first appearance of the canid *Hesperocyon* cf. *H. gregarius* in the Duchesnean Lac Pelletier Lower Fauna of the Cypress Hills Formation, Saskatchewan, Canada [50], [114] (N.B. an earlier study [7] noted the first appearance date of ca. 43 Ma for *Daphoenus* and *Hesperocyon*, but the derivation of this date is unclear; no unambiguous occurrence of a canid or amphicyonid is currently known prior to the Duchesnean NALMA). A less-secure minimum divergence date of 40 Ma may instead be proposed for the same cladistic topology based on the occurrence of an unidentified nimravid in the Hancock Mammal Quarry of the Clarno Formation, Oregon, below a welded tuff layer in the Member A of the John Day Formation, which has yielded ^40^Ar/^39^Ar dates of 39.5–40.0 Ma [115], [116].
2. If “*Miacis*” *sylvestris* is located inside the Carnivora, the conservative minimum divergence date will be approximately 47 Ma based on its first appearance near the top of the Upper Blacks Fork Member [3], [113] of the Bridger Formation, Wyoming, below the Henrys Fork Tuff in the overlaying Twin Buttes Member, which has yielded a ^40^Ar/^39^Ar date of 46.9±0.2 Ma [113], [117].

Comparison of these minimum dates of caniform-feliform divergence with divergence-date estimates reported in molecular phylogenetic studies is hampered by the fact that most of the published molecular-clock estimates depend on a fossil constraint placed on this very node of interest. The problem is further complicated by the frequent selection of fossil constraints [27], [28], [30], [31], [36]–[38] using taxonomic classifications that either do not distinguish between crown and stem groups or implicitly assume the inclusion of all carnivoramorphans in the crown-group Carnivora [118], [119]. The few estimates that do not depend on a fossil constraint placed on the node of carnivoran origin are widely divergent, ranging from 63±2 (standard error) Ma [10], [11] to 46±6 (standard error) Ma [120]. The accuracies of both of these estimates may be questioned, however, because of the choice of *Procynodictis vulpiceps* to constrain the base of Canidae in the former case [10], [11] (following [118], but not supported by the present or past cladistic analyses [6]), and because of the reliance on a single fossil calibration point (310 Ma for the synapsids-diapsid split) in the latter case [120] in deriving the time scale for a vertebrate phylogeny (cf. [121], [122]).

*Lycophocyon hutchisoni* sheds an additional light on the morphological evolution of caniforms surrounding the origin of the crown-group Canoidea. In the middle to late Eocene, early caniforms such as *L. hutchisoni*, *Cynodictis* (e.g. *C. lacustris*), and *Daphoenus* (e.g. *D. lambei*) were generally characterized by M1s with labially-extended parastylar regions, diminutive M3s, relatively closed m1 trigonids with well-developed metaconids, and absence of well-ossified entotympanics that were firmly fused to the basicranium. With minor modifications, the features of M1 and m1 were inherited by the most-basal canoids from the late Eocene to the early Oligocene, such as *Prohesperocyon wilsoni* (putatively the most primitive, though not the earliest-known, stem canid [50]), *Mustelavus priscus*, and *Amphicticeps shackelfordi* [47]. On the other hand, loss of M3 and development of well-ossified entotympanics seem to be closely associated with the canoid origin sometime before 38 Ma; this implies that the same transformations independently took place among early feliforms. Apparently very early in the history of the canid lineage, the parastylar extension of M1 was suppressed, the metaconid of m1 was substantially reduced, the m1 trigonid became quite open (i.e., much longer than wide), and a partial septum was formed inside the ossified auditory bulla, as seen in the earliest-known canid *Hesperocyon gregarius*. Similar modifications of M1 and m1 are seen within early arctoids (e.g. “amphicynodonts”) and amphicyonids, respectively.

Consideration of the biogeographic and ecological context of the carnivoran origin depends on a clear understanding of the phylogenetic relationships of the early carnivorans and their close carnivoramorphan relatives outside the crown group. Cladistic analyses of wider arrays of Paleogene carnivoramorphans are thus awaited.

## Materials and Methods

All currently-known specimens of *Lycophocyon hutchisoni* are housed at the University of California Museum of Paleontology and the San Diego Natural History Museum. A list of comparative specimens directly examined by the author is provided in Appendix S1. Skeletal comparisons with extant carnivorans are based on the author's direct observation of modern specimens. The taxonomic classification of extant carnivorans follows Wilson and Reeder [123].

### Anatomical Terminology and Measurements

The anatomical terminology used in this paper follows primarily: Mac Intyre [124], Van Valen [125], Flynn and Galiano [5], and Heinrich et al. [126] for dentition; Wang and Tedford [6] for basicranium; and Gingerich [62] and Heinrich and Rose [59] for postcrania. All measurements were taken with digital calipers with the accuracy of 0.01 mm, and are reported to the nearest 0.1 mm. Dental measurements follow Gingerich [62], and measurements of humerus and ulna follow Meachen-Samuels and Van Valkenburgh [127].

### Statistical Comparisons of Size Variation

The comparative samples consisted of 27 specimens of the earliest-known canid *Hesperocyon gregarius* from the late Eocene to early Oligocene (Chadronian to Whitneyan NALMA) of the northern and central Great Plains, 51 specimens of *Urocyon cinereoargenteus townsendi* from California, U.S.A., and 29 specimens of *Martes pennanti columbiana* from British Columbia, Canada. All measurements are reported in Appendix S2. For the fossil taxa, only the specimens that could be confidently assigned to separate individuals were measured to avoid data duplication. Both the differences among the sample means (not exceeding an order of magnitude) and the percent measurement errors (0.8 to 5.5%) are sufficiently small for proper comparisons of CVs (cf. [128]).

Because the conventional *F*-ratio test is sensitive to non-normal distribution of data [129], the randomization procedure of Lockwood et al. [70] was adopted for the present analysis. From each comparative sample, 10,000 bootstrap replicates [130] of 9 m1L measurements (to make the subsample size equal to the sample size of *Lycophocyon hutchisoni*) were produced, and the CV was calculated for each replicate. Finally, the frequency distribution of 10,000 CVs was compared to the CV of *L. hutchisoni*; if the latter fell outside the bias-corrected 95% confidence interval [131] of the former, the sample of *L. hutchisoni* was considered to be significantly more (or less) variable than that of the comparative taxon. All computations were performed in the R programming environment Version 2.10.1 for Windows [132].

### Dietary Inference

The body weight of the individual represented by the holotype UCMP 85202 was estimated using a rescaled version of the least-squares regression equation of Van Valkenburgh [133]: *LBW* = 2.97 ln(*m1L*)+1.68, where *LBW* is the natural log-transformed body weight in grams. This equation was derived from data on extant placental carnivorans (69 species) and marsupial carnivores (2 species), including representatives of all carnivoran families other than the Herpestidae and Eupleridae, and ranging in body weight from roughly 140 g to 400 kg. A more accurate body-weight estimate based on the condylobasal length of a cranium is available for the referred specimen SDSNH 107465 (*LBW* = 3.13 ln(105.3 (mm))−5.96 = 5.50×10^3^ (g); rescaled equation from [133]), but it is practically identical to the estimate obtained for UCMP 85202. Body weight estimates based on cross-sectional areas of proximal limb bones would be ideal but are not possible with the available specimens, in which diaphyses are crushed.

The dietary inference for *Lycophocyon hutchisoni* is based on a linear discriminant analysis of estimated body weight and craniodental morphology. Data on the diet (divided into three groups: carnivorous, insectivorous, and omnivorous/durophagous), body weight, and ecomorphological indices of 82 extant species of small to medium-sized carnivorans (body weight ≤30 kg) were adopted from Friscia et al. [71] (*Poiana richardsonii* was excluded from the data set because of a missing datum). To generate a set of classification functions for the prediction of the diet of *L. hutchisoni*, all possible subsets of 10 predictor variables (consisting of log-transformed body weight and 9 variables that were shown by Friscia et al. [71] to differ significantly among the dietary groups) were subjected to the linear discriminant analysis, whereby the success rate of jackknife re-classification was evaluated for each subset of variables.

A set of dietary classification functions with 6 predictor variables was then chosen based on the highest overall jackknife re-classification success rate of 88%, with correct dietary identification of 93% of the carnivores, 93% of the insectivores, and 71% of the omnivores/hard-object feeders in the data set (see Table 3 for additional information and abbreviations). The first and second discriminant functions are given as:
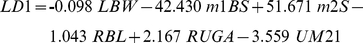


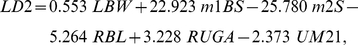
and account for 69% and 31% of the between-group variance, respectively.

The dietary classification of *Lycophocyon hutchisoni* is based on measurements of the holotype UCMP 85202, from which the following values were obtained: *LBW* = 8.73, *RBL* = 0.682, *RUGA* = 1.068, *M1BS* = 0.074, *M2S* = 0.058, *UM21* = 0.638. The linear discriminant analysis was performed with the MASS package Version 7.3-5 [134] in the R programming environment.

### Cladistic Analysis

#### Character matrix data

The morphological character matrix of Wesley-Hunt and Flynn [7] and additional data from subsequent studies [57], [72]–[74] were adopted for the cladistic analysis in this paper. The numbering of characters and the treatment of Character 40 as an additive character (all others are non-additive) follow these previous studies, and the identification of operational taxonomic units (OTUs) represented by referred specimens (indicated by “cf.”) follows Polly et al. [74]. For the present analysis, Character 43 was eliminated (cf. [57]), and the OTUs originally identified [7] as *Hyaenodon cruentus*, *Prohesperocyon wilsoni*, and *Protictis schaffi* are considered to represent *Hyaenodon horridus* [135], “*Miacis*” *gracilis* (cf. [57]; “*M.*” *gracilis* is possibly a junior synonym of *Procynodictis vulpiceps* [50], [51]), and *Viverravus politus* [136], respectively. The matrix data for *Lycophocyon hutchisoni* are based on the holotype UCMP 85202 and paratypes UCMP 170713, SDSNH 107443, SDSNH 107444, and SDSNH 107659. Character matrix data for the following additional taxa were collected by the author and were included in the analysis: *Amphicticeps shackelfordi*, *Broiliana nobilis*, *Daphoenus*, *Mustelavus priscus*, *Plesictis genettoides*, and *Pseudobassaris riggsi* (see Appendix S1 for a list of the specimens examined). Of these, the data for *Daphoenus* replaced those for the composite amphicyonid OTU in the previous studies [7], [57], [72]–[74]. The composite OTU for *Daphoenus* is represented by specimens referred to *D. hartshornianus*, *D. vetus*, and undetermined species of the genus (most likely *D. hartshornianus* or *D. vetus*); the two currently-recognized species are skeletally quite similar except for size [53] and difficult to distinguish when comparing large individuals of *D. hartshornianus* with small individuals of *D. vetus* [53], [137], making their specific distinction questionable [137]. The state of Character 89 (size of baculum) for *Daphoenus* was determined based on a published account and figures of *D. vetus* (CM 492) [56]. Appendix S3 contains the complete character matrix analyzed for the present study.

#### Analytical procedure

Parsimony analysis was conducted with the program TNT Version 1.1 [138], [139] for (1) the full data set of 98 characters and 50 OTUs, in which *Leptictis dakotensis*, *Erinaceus concolor*, and *Echinosorex gymnura* were placed in the outgroup and (2) its subset consisting of 33 OTUs that represent taxa known from the Paleogene Period. The most-parsimonious trees were heuristically searched for using the “traditional search” function of the program with the tree bisection and reconnection algorithm and 3,000 random-addition sequence replicates. The nodal support for the consensus tree was assessed in two ways: (1) the Bremer support value for each node [140] was determined by step-wise inspection of the consensus of suboptimal trees in TNT and, for well-supported groups, using the Bremer.run script of Goloboff et al. [139] (available at tnt.insectmuseum.org/images/0/08/Bremer.run); (2) using 1,000 pseudo-replicates of the character matrix, bootstrap support values were obtained to evaluate the effect of differential weighting of characters [141].The ensemble consistency index (CI) [142] and ensemble retention index (RI) [143] for the most-parsimonious trees were calculated using the program Mesquite Version 6.72 [144]. Synapomorphies were identified by the optimization function of TNT and the parsimony reconstruction of ancestral character states using Mesquite.

### Nomenclatural Acts

The electronic version of this document does not represent a published work according to the International Code of Zoological Nomenclature (ICZN), and hence the nomenclatural acts contained in the electronic version are not available under that Code from the electronic edition. Therefore, a separate edition of this document was produced by a method that assures numerous identical and durable copies, and those copies were simultaneously obtainable (from the publication date noted on the first page of this article) for the purpose of providing a public and permanent scientific record, in accordance with Article 8.1 of the Code. The separate print-only edition is available on request from PLoS by sending a request to PLoS ONE, Public Library of Science, 1160 Battery Street, Suite 100, San Francisco, CA 94111, U.S.A., along with a check for $10 (to cover printing and postage) payable to “Public Library of Science”.

In addition, this published work and the nomenclatural acts it contains have been registered in ZooBank, the proposed online registration system for the ICZN. The ZooBank LSIDs (Life Science Identifiers) can be resolved and the associated information viewed through any standard web browser by appending the LSID to the prefix “http://zoobank.org/”. The LSID for this publication is: urn:lsid:zoobank.org:pub:28287C88-C386-4C75-894E-C6060AE9B7E2.

## Supporting Information

Appendix S1 List of comparative specimens examined.
(DOC)

Appendix S2 Measurements of the lower first molars of *Lycophocyon hutchisoni*, *Hesperocyon gregarius*, *Urocyon cinereoargenteus townsendi*, and *Martes pennanti columbiana* used for the analysis of size variation.
(DOC)

Appendix S3 Nexus file for the program Mesquite [144] containing the character matrix for the cladistic analysis, most-parsimonious trees for the full set of 50 OTUs (labeled as MPT50-1 through MPT50-132) and the subset consisting of 33 OTUs (labeled as MPT33-1 through MPT33-32), and the strict consensus for each set of taxa (labeled Consensus50 and Consensus33, respectively). For the full set of 50 OTUs, only the trees with *Leptictis dakotensis* as the primary outgroup taxon are shown; as noted in the Results, the choice of either *Thinocyon* sp. or *Hyaenodon horridus* as the primary outgroup taxon does not affect the topology of non-viverravid carnivoramorphans. The character numbers of Wesley-Hunt and Flynn [7] are denoted with the prefix “whf.” The parsimony reconstruction of ancestral character states can be viewed in the Tree Window of Mesquite.
(NEX)
