# Supplementary material for: A New Basal Caniform (Mammalia: Carnivora) from the Middle Eocene of North America and Remarks on the Phylogeny of Early Carnivorans
Source: PLoS One. 2011 Sep 14;6(9):e24146. doi: 10.1371/journal.pone.0024146 (PMC3173397; doi:10.1371/journal.pone.0024146)
Supplement: Appendix S1 — List of comparative specimens examined. (DOC) [file pone.0024146.s001.doc]

**Appendix S1.** List of comparative specimens examined.

| **Taxon** | **Specimen** | **References** |
| --- | --- | --- |
| *Hesperocyon gregarius* | UCMP 126095 | Wang, 1993 |
| *Hesperocyon gregarius* | UCMP 31878 |  |
| “*Miacis*” *gracilis* | CM 11900 (holotype) | Clark, 1939 |
| *“Miacis” cognitus* | AMNH FM 127171^2^ | Gustafson, 1986 |
| *Tapocyon robustus* | SDSNH 36000 | Wesley and Flynn, 2003 |
| *Amphicticeps shackelfordi* | AMNH FM 19010 (holotype)^1^ | Wang et al., 2005 |
| *Amphicticeps shackelfordi* | UCMP 79943^1,3^ |  |
| *Broiliana nobilis* | AMNH FM 108381^1,4^ | Dehm, 1950 |
| *Broiliana nobilis* | AMNH FM 108382^1,5^ | Dehm, 1950; Wolsan, 1993; Wang et al., 2005 |
| *Daphoenus hartshornianus* | AMNH FM 9757^1^ | Hough, 1948 |
| *Daphoenus hartshornianus* | AMNH FM 12450^1^ | Hough, 1948 |
| *Daphoenus hartshornianus* | YPM PU 11424^1^ | Scott, 1898 |
| *Daphoenus* sp. | UCMP 27561 |  |
| *Daphoenus* sp. | AMNH FM 63330^1^ |  |
| *Daphoenus* sp. | AMNH FM 76529^1^ |  |
| *Paradaphoenus minimus* | AMNH FM 39099 | Hough, 1948; Hunt, 2001 |
| *Mustelavus priscus* | YPM PU 13775^1^ | Scott and Jepsen 1936; Clark, 1937; Hough, 1948b; Schmidt-Kittler, 1981; Wang et al., 2005 |
| *Mustelavus priscus* | AMNH FM 129168^1^ | Wang et al., 2005 |
| *Plesictis genettoides* | AMNH FM 11001^1^ | Hough, 1948b; Schmidt-Kittler, 1981; Wolsan, 1993 |
| *Pseudobassaris riggsi* | YPM PU 11455^1^ (holotype) | Riggs, 1898; Pohle, 1917; Hough, 1948b; Schmidt-Kittler, 1981; Wolsan and Lange-Badré, 1996; Wang et al., 2005 |
| *Urocyon cinereoargenteus* | UCMP 58805 |  |
| *Vulpes vulpes* | UCMP 86007 |  |
| *Vulpes vulpes* | UCMP 117575 |  |
| *Otocyon megalotis* | MVZ 118422 |  |
| *Ursus americanus* | UCMP 8816 |  |
| *Ursus americanus* | UCMP 8819 |  |
| *Gulo gulo* | MVZ 184100 |  |
| *Martes pennanti* | MVZ 23668 |  |
| *Martes pennanti* | MVZ 24740 |  |
| *Martes pennanti* | MVZ 29812 |  |
| *Taxidea taxus* | UCMP 122655 |  |
| *Nasua narica* | MVZ 98257 |  |
| *Procyon lotor* | UCMP 122695 |  |
| *Procyon lotor* | UCMP 122696 |  |
| *Potos flavus* | MVZ 98273 |  |
| *Potos flavus* | MVZ 98274 |  |
| *Ailurus fulgens* | CAS 14855 |  |
| *Felis rufus* | UCMP 122623 |  |
| *Felis rufus* | UCMP 122625 |  |
| *Arctictis binturong* | MVZ 136247 |  |
| *Paradoxurus hermaphroditus* | MVZ 186575 |  |
| *Atilax paludinosus* | MVZ 118462 |  |

^1^Specimens examined for the cladistic analysis in the present study. ^2^Cast of holotype TMM 40209-200. ^3^Cast of holotype AMNH 19010. ^4^Cast of BSP 1937 II 13191. ^5^Cast of holotype BSP 1937 II 13524. **Institutional abbreviations**: **AMNH**, American Museum of Natural History (New York, New York, U.S.A.); **CAS**, California Academy of Sciences (San Francisco, California, U.S.A.); **CM**, Carnegie Museum of Natural History (Pittsburgh, Pennsylvania, U.S.A.); **MVZ**, Museum of Vertebrate Zoology, University of California (Berkeley, California, U.S.A.); **SDSNH**, San Diego Natural History Museum (San Diego, California, U.S.A.); **UCMP**, University of California Museum of Paleontology (Berkeley, California, U.S.A.); **YPM**, Yale Peabody Museum of Natural History (New Haven, Connecticut, U.S.A.).

**References**

Clark J (1937) The stratigraphy and paleontology of the Chadron Formation in the Big Badlands of South Dakota. Annals of the Carnegie Museum 25: 261-350.

Clark J (1939) *Miacis gracilis*, a new carnivore from the Uinta Eocene. Annals of the Carnegie Museum 27: 349-371.

Dehm R (1950) Die Raubtiere aus dem Mittel-Miocän (Burgidalium) von Wintershof-West bei Eichstätt in Bayern. Abhandlungen der Bayerischen Akademie der Wissenschaften, Mathematisch-naturwissenschaftliche Klasse 58: 1-141.

Gustafson EP (1986) Carnivorous mammals of the late Eocene and early Oligocene of Trans-Pecos Texas. Bulletin of the Texas Memorial Museum: 1-66.

Hunt RM, Jr. (2001) Small Oligocene amphicyonids from North America (*Paradaphoenus*, Mammalia, Carnivora). American Museum Novitates 3331: 1-20.

Hough JR (1948) A systematic revision of *Daphoenus* and some allied genera. Journal of Paleontology 22: 573-600.

Hough JR (1948b) The auditory regions in some members of the Procyonidae, Canidae, and Ursidae: its significance in the phylogeny of the Carnivora. Bulletin of the American Museum of Natural History 92: 67-118.

Pohle H (1917) *Pseudobassaris riggsi*, gen. nov. spec. nov. für *Amphictis* spec. Riggs. Sitzungsberichte der Gesellschaft Naturforschender Freunde zu Berlin 1917: 403–411.

Riggs ES (1898) On the skull of *Amphictis*. American Journal of Science 5: 257-259.

Schmidt-Kittler N (1981) Zur Stammesgeschichte der marderverwandten Raubtiergruppen (Musteloidea, Carnivora). Ecologae geologicae Helvetiae 74: 753-801.

Scott WB (1898) Notes on the Canidae of the White River Oligocene. Transactions of the American Philosophical Society 19: 325-416.

Scott WB, Jepsen GL (1936) The mammalian fauna of the White River Oligocene: part I, Insectivora and Carnivora. Transactions of the American Philosophical Society 28: 1-153.

Wang X (1993) Transformation from plantigrady to digitigrady: functional morphology of locomotion in *Hesperocyon* (Canidae: Carnivora). American Museum Novitates 3069: 1-23.

Wang X, McKenna MC, Dashzeveg D (2005) *Amphicticeps* and *Amphicynodon* (Arctoidea, Carnivora) from Hsanda Gol Formation, central Mongolia and phylogeny of basal arctoids with comments on zoogeography. American Museum Novitates 3483: 1-57.

Wesley GD, Flynn JJ (2003) A revision of *Tapocyon* (Carnivoramorpha), including analysis of the first cranial specimens and identification of a new species. Journal of Paleontology 77: 769-783.

Wolsan M (1993) Phylogeny and classification of early European Mustelida (Mammalia: Carnivora). Acta Theriologica 38: 345-384.

Wolsan M, Lange-Badré B (1996) An arctomorph carnivoran skull from the Phosphorites du Quercy and the origin of procyonids. Acta Palaeontologica Polonica 41: 277-298.
