# Supplementary material for: A New Basal Caniform (Mammalia: Carnivora) from the Middle Eocene of North America and Remarks on the Phylogeny of Early Carnivorans
Source: PLoS One. 2011 Sep 14;6(9):e24146. doi: 10.1371/journal.pone.0024146 (PMC3173397; doi:10.1371/journal.pone.0024146)
Supplement: Appendix S2 — Measurements of the lower first molars of Lycophocyon hutchisoni, Hesperocyon gregarius, Urocyon cinereoargenteus townsendi, and Martes pennanti columbiana used for the analysis of size variation. (DOC) [file pone.0024146.s002.doc]

**Appendix S2.** Measurements of the lower first molars of *Lycophocyon hutchisoni*, *Hesperocyon gregarius*, *Urocyon cinereoargenteus townsendi*, and *Martes pennanti columbiana* used in the analysis of size variation

| **Institution** | **Specimen** | **Taxon** | **Sex^1^** | **m1L (mm)** |
| --- | --- | --- | --- | --- |
| SDSNH | 107450 | *Lycophocyon hutchisoni* | U | 9.1 |
| SDSNH | 107447 | *Lycophocyon hutchisoni* | U | 9.4 |
| SDSNH | 107448 | *Lycophocyon hutchisoni* | U | 9.5 |
| SDSNH | 107455 | *Lycophocyon hutchisoni* | U | 9.5 |
| SDSNH | 107442 | *Lycophocyon hutchisoni* | U | 10.1 |
| SDSNH | 92094 | *Lycophocyon hutchisoni* | U | 10.7 |
| UCMP | 170713 | *Lycophocyon hutchisoni* | U | 9.6 |
| UCMP | 85202 | *Lycophocyon hutchisoni* | U | 10.7 |
| UCMP | 313994 | *Lycophocyon hutchisoni* | U | 10.3 |
| AMNH | FM 1004 | *Hesperocyon gregarius* | U | 8.4 |
| AMNH | FM 1472 | *Hesperocyon gregarius* | U | 8.7 |
| AMNH | FM 5297 | *Hesperocyon gregarius* | U | 9.6 |
| AMNH | FM 5298 | *Hesperocyon gregarius* | U | 9.0 |
| AMNH | FM 5299 | *Hesperocyon gregarius* | U | 9.0 |
| AMNH | FM 5307 | *Hesperocyon gregarius* | U | 9.5 |
| AMNH | FM 5315 | *Hesperocyon gregarius* | U | 9.0 |
| AMNH | FM 50244 | *Hesperocyon gregarius* | U | 9.2 |
| AMNH | FM 50251 | *Hesperocyon gregarius* | U | 9.0 |
| AMNH | FM 50288 | *Hesperocyon gregarius* | U | 10.0 |
| AMNH | FM 82918 | *Hesperocyon gregarius* | U | 9.3 |
| AMNH | FM 85884 | *Hesperocyon gregarius* | U | 10.0 |
| LACM | CIT 621 | *Hesperocyon gregarius* | U | 9.7 |
| LACM | CIT 1400 | *Hesperocyon gregarius* | U | 9.0 |
| LACM | CIT 1526 | *Hesperocyon gregarius* | U | 9.3 |
| UCMP | 31878 | *Hesperocyon gregarius* | U | 9.2 |
| UCMP | 31879 | *Hesperocyon gregarius* | U | 9.3 |
| UCMP | 32946 | *Hesperocyon gregarius* | U | 9.7 |
| UCMP | 77162 | *Hesperocyon gregarius* | U | 9.6 |
| UCMP | 77167 | *Hesperocyon gregarius* | U | 9.2 |
| UCMP | 113702 | *Hesperocyon gregarius* | U | 9.7 |
| UCMP | 113703 | *Hesperocyon gregarius* | U | 9.8 |
| UCMP | 113704 | *Hesperocyon gregarius* | U | 10.2 |
| UCMP | 133968 | *Hesperocyon gregarius* | U | 9.9 |
| UCMP | 311062 | *Hesperocyon gregarius* | U | 9.7 |
| UCMP | 323676 | *Hesperocyon gregarius* | U | 8.9 |
| UCMP | 323677 | *Hesperocyon gregarius* | U | 8.8 |
| MVZ | 3615 | *Urocyon cinereoargenteus townsendi* | F | 12.1 |
| MVZ | 3616 | *Urocyon cinereoargenteus townsendi* | M | 12.7 |
| MVZ | 3619 | *Urocyon cinereoargenteus townsendi* | F | 11.8 |
| MVZ | 4097 | *Urocyon cinereoargenteus townsendi* | M | 11.4 |
| MVZ | 8894 | *Urocyon cinereoargenteus townsendi* | M | 12.8 |
| MVZ | 8967 | *Urocyon cinereoargenteus townsendi* | M | 11.4 |
| MVZ | 8968 | *Urocyon cinereoargenteus townsendi* | F | 12.1 |
| MVZ | 9532 | *Urocyon cinereoargenteus townsendi* | M | 13.5 |
| MVZ | 9533 | *Urocyon cinereoargenteus townsendi* | U | 12.5 |
| MVZ | 11735 | *Urocyon cinereoargenteus townsendi* | M | 12.9 |
| MVZ | 11736 | *Urocyon cinereoargenteus townsendi* | F | 12.7 |
| MVZ | 19100 | *Urocyon cinereoargenteus townsendi* | M | 12.0 |
| MVZ | 19155 | *Urocyon cinereoargenteus townsendi* | M | 12.3 |
| MVZ | 19707 | *Urocyon cinereoargenteus townsendi* | M | 11.9 |
| MVZ | 19710 | *Urocyon cinereoargenteus townsendi* | F | 11.5 |
| MVZ | 20751 | *Urocyon cinereoargenteus townsendi* | U | 11.7 |
| MVZ | 21549 | *Urocyon cinereoargenteus townsendi* | M | 12.6 |
| MVZ | 21551 | *Urocyon cinereoargenteus townsendi* | M | 12.9 |
| MVZ | 21552 | *Urocyon cinereoargenteus townsendi* | F | 12.5 |
| MVZ | 21555 | *Urocyon cinereoargenteus townsendi* | F | 12.6 |
| MVZ | 22106 | *Urocyon cinereoargenteus townsendi* | M | 12.8 |
| MVZ | 22107 | *Urocyon cinereoargenteus townsendi* | M | 11.5 |
| MVZ | 23507 | *Urocyon cinereoargenteus townsendi* | M | 11.5 |
| MVZ | 23691 | *Urocyon cinereoargenteus townsendi* | F | 12.4 |
| MVZ | 23692 | *Urocyon cinereoargenteus townsendi* | M | 11.5 |
| MVZ | 24418 | *Urocyon cinereoargenteus townsendi* | F | 11.6 |
| MVZ | 24419 | *Urocyon cinereoargenteus townsendi* | F | 12.0 |
| MVZ | 24741 | *Urocyon cinereoargenteus townsendi* | M | 12.0 |
| MVZ | 24742 | *Urocyon cinereoargenteus townsendi* | F | 12.1 |
| MVZ | 28792 | *Urocyon cinereoargenteus townsendi* | M | 11.7 |
| MVZ | 28793 | *Urocyon cinereoargenteus townsendi* | F | 11.8 |
| MVZ | 37482 | *Urocyon cinereoargenteus townsendi* | U | 12.1 |
| MVZ | 84281 | *Urocyon cinereoargenteus townsendi* | M | 13.1 |
| MVZ | 90451 | *Urocyon cinereoargenteus townsendi* | F | 11.8 |
| MVZ | 90712 | *Urocyon cinereoargenteus townsendi* | F | 11.7 |
| MVZ | 96223 | *Urocyon cinereoargenteus townsendi* | M | 12.3 |
| MVZ | 101178 | *Urocyon cinereoargenteus townsendi* | M | 12.8 |
| MVZ | 101500 | *Urocyon cinereoargenteus townsendi* | M | 12.1 |
| MVZ | 101770 | *Urocyon cinereoargenteus townsendi* | M | 12.4 |
| MVZ | 102296 | *Urocyon cinereoargenteus townsendi* | M | 11.6 |
| MVZ | 108077 | *Urocyon cinereoargenteus townsendi* | M | 12.1 |
| MVZ | 115606 | *Urocyon cinereoargenteus townsendi* | M | 12.3 |
| MVZ | 125977 | *Urocyon cinereoargenteus townsendi* | F | 12.5 |
| MVZ | 132529 | *Urocyon cinereoargenteus townsendi* | M | 12.3 |
| MVZ | 135437 | *Urocyon cinereoargenteus townsendi* | M | 12.3 |
| MVZ | 135438 | *Urocyon cinereoargenteus townsendi* | F | 12.2 |
| MVZ | 140631 | *Urocyon cinereoargenteus townsendi* | M | 11.3 |
| MVZ | 149565 | *Urocyon cinereoargenteus townsendi* | M | 12.0 |
| MVZ | 149768 | *Urocyon cinereoargenteus townsendi* | M | 12.2 |
| MVZ | 149773 | *Urocyon cinereoargenteus townsendi* | F | 11.8 |
| MVZ | 185217 | *Urocyon cinereoargenteus townsendi* | M | 12.6 |
| CAS | 22153 | *Martes pennanti columbiana* | M | 14.3 |
| MVZ | 41119 | *Martes pennanti columbiana* | U | 12.4 |
| MVZ | 43613 | *Martes pennanti columbiana* | U | 14.2 |
| MVZ | 43614 | *Martes pennanti columbiana* | U | 12.6 |
| MVZ | 43615 | *Martes pennanti columbiana* | U | 12.3 |
| MVZ | 43616 | *Martes pennanti columbiana* | U | 12.0 |
| MVZ | 43617 | *Martes pennanti columbiana* | U | 12.1 |
| MVZ | 43618 | *Martes pennanti columbiana* | U | 14.3 |
| MVZ | 43619 | *Martes pennanti columbiana* | U | 12.5 |
| MVZ | 43621 | *Martes pennanti columbiana* | U | 12.4 |
| MVZ | 43622 | *Martes pennanti columbiana* | U | 12.0 |
| MVZ | 43623 | *Martes pennanti columbiana* | U | 12.2 |
| MVZ | 43624 | *Martes pennanti columbiana* | U | 14.0 |
| MVZ | 43626 | *Martes pennanti columbiana* | U | 12.2 |
| MVZ | 43627 | *Martes pennanti columbiana* | U | 14.1 |
| MVZ | 44531 | *Martes pennanti columbiana* | U | 14.5 |
| MVZ | 44532 | *Martes pennanti columbiana* | U | 12.4 |
| MVZ | 44533 | *Martes pennanti columbiana* | U | 13.8 |
| MVZ | 44534 | *Martes pennanti columbiana* | U | 12.3 |
| MVZ | 44536 | *Martes pennanti columbiana* | F | 11.9 |
| MVZ | 44537 | *Martes pennanti columbiana* | U | 12.7 |
| MVZ | 44538 | *Martes pennanti columbiana* | U | 13.9 |
| MVZ | 44539 | *Martes pennanti columbiana* | U | 12.2 |
| MVZ | 44540 | *Martes pennanti columbiana* | U | 12.1 |
| MVZ | 44541 | *Martes pennanti columbiana* | U | 14.3 |
| MVZ | 44542 | *Martes pennanti columbiana* | U | 14.0 |
| MVZ | 44543 | *Martes pennanti columbiana* | U | 12.1 |
| MVZ | 54934 | *Martes pennanti columbiana* | U | 12.2 |
| MVZ | 54935 | *Martes pennanti columbiana* | U | 12.1 |

^1^Sex is denoted as F (female), M (male), or U (undetermined). **Institutional abbreviations**: **AMNH**, American Museum of Natural History (New York, New York, U.S.A.); **CAS**, California Academy of Sciences (San Francisco, California, U.S.A.); **LACM**, Natural History Museum of Los Angeles County (Los Angeles, California, U.S.A.); **MVZ**, Museum of Vertebrate Zoology, University of California, Berkeley (Berkeley, California, U.S.A.); **SDSNH**, San Diego Natural History Museum (San Diego, California, U.S.A.); **UCMP**, University of California Museum of Paleontology (Berkeley, California, U.S.A.).
